# Bridging the Homogeneous-Heterogeneous Divide: Modeling Spin for Reactivity in Single Atom Catalysis

**DOI:** 10.3389/fchem.2019.00219

**Published:** 2019-04-16

**Authors:** Fang Liu, Tzuhsiung Yang, Jing Yang, Eve Xu, Akash Bajaj, Heather J. Kulik

**Affiliations:** ^1^Department of Chemical Engineering, Massachusetts Institute of Technology, Cambridge, MA, United States; ^2^Department of Materials Science and Engineering, Massachusetts Institute of Technology, Cambridge, MA, United States

**Keywords:** density functional theory, catalysis, single atom catalysis, spin state crossover, transition metal chemistry

## Abstract

Single atom catalysts (SACs) are emergent catalytic materials that have the promise of merging the scalability of heterogeneous catalysts with the high activity and atom economy of homogeneous catalysts. Computational, first-principles modeling can provide essential insight into SAC mechanism and active site configuration, where the sub-nm-scale environment can challenge even the highest-resolution experimental spectroscopic techniques. Nevertheless, the very properties that make SACs attractive in catalysis, such as localized *d* electrons of the isolated transition metal center, make them challenging to study with conventional computational modeling using density functional theory (DFT). For example, Fe/N-doped graphitic SACs have exhibited spin-state dependent reactivity that remains poorly understood. However, spin-state ordering in DFT is very sensitive to the nature of the functional approximation chosen. In this work, we develop accurate benchmarks from correlated wavefunction theory (WFT) for relevant octahedral complexes. We use those benchmarks to evaluate optimal DFT functional choice for predicting spin state ordering in small octahedral complexes as well as models of pyridinic and pyrrolic nitrogen environments expected in larger SACs. Using these guidelines, we determine Fe/N-doped graphene SAC model properties and reactivity as well as their sensitivities to DFT functional choice. Finally, we conclude with broad recommendations for computational modeling of open-shell transition metal single-atom catalysts.

## Introduction

**Figure 1:**
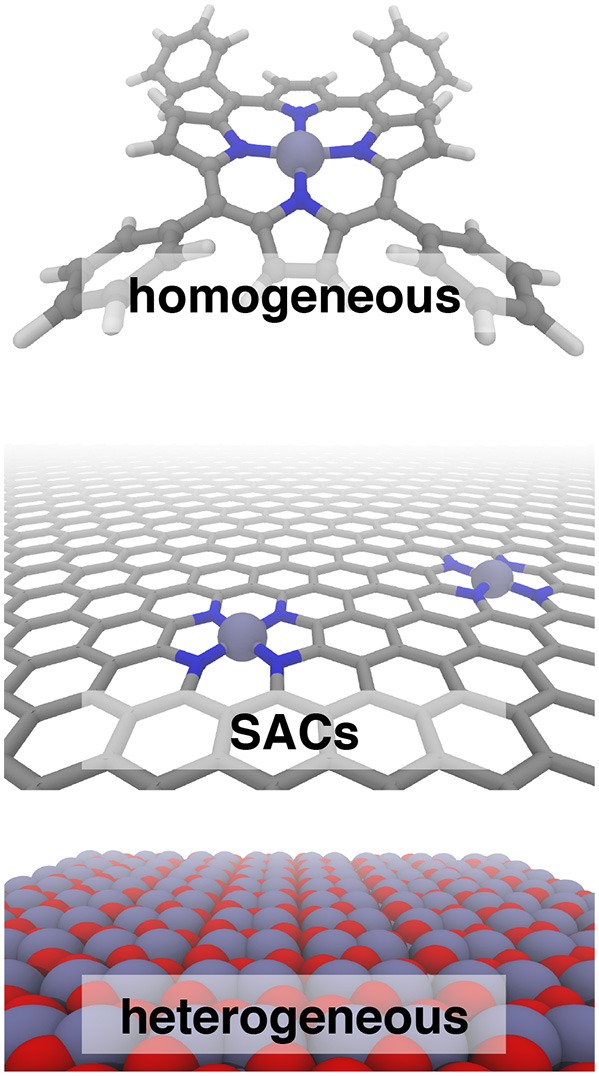
Iron catalysts in three classes: homogeneous tetraphenyl porphyrin **(Top)**, N-doped graphene single atom catalysts (SACs, **Middle**), and heterogeneous hematite **(Bottom)**. In all cases, carbon is shown in gray, nitrogen in blue, iron in purple, and oxygen in red.

Single atom catalysts (SACs) (Yang et al., 2013) are emergent catalytic materials (Yang et al., 2013; Liang et al., 2015, 2017) that have the promise of merging the scalability of heterogeneous catalysts with the high activity and atom economy of homogeneous catalysts, but the reactivity of SACs is poorly understood (Figure 1). Fe/N-doped graphene SACs have been demonstrated for critical transformations such as selective hydrocarbon oxidation (Liu et al., 2017), including ambient methane to methanol conversion (Cui et al., 2018), and as non-Pt oxygen reduction reaction (ORR) electrocatalysts (Li et al., 2016; Chen et al., 2017; Yang et al., 2018). The short-lived, variable SAC active sites that are fundamentally sub-nm-scale challenge the resolution of spectroscopic techniques (Fei et al., 2015; Wang and Zhang, 2016), making first-principles modeling essential to mechanistic study.

For these emerging catalysts, changing synthesis (Liu et al., 2017) or reaction (Li et al., 2016; Zitolo et al., 2017) conditions changes the distribution of SAC coordination geometries, and the most reactive species for key reactions (e.g., ORR or selective partial hydrocarbon oxidation) remain under debate (Zitolo et al., 2015; Zhu et al., 2017; Yang et al., 2018). In selective partial hydrocarbon oxidation, spin-state-dependent reactivity of Fe/N-doped graphene SACs has been observed, with an intermediate, five-coordinate Fe(III)-N-C catalyst more reactive and selective (Liu et al., 2017) than low-spin or high-spin Fe active sites with four- or six-fold coordination. The fundamental source of this spin-state dependent reactivity remains unknown. In SAC electrocatalysts (Fei et al., 2015; Qiu et al., 2015; Zitolo et al., 2015, 2017; Back et al., 2017; Chen et al., 2017; Cheng et al., 2017; Zhang et al., 2017a,b; Zhu et al., 2017; Gao et al., 2018; Jiang et al., 2018; Wang et al., 2018; Zhang et al., 2018), changes in applied potential (e.g., in ORR) have been suggested to change the Fe SAC active site, possibly through a change in spin state (Zitolo et al., 2017).

Although perhaps surprising in the context of heterogeneous catalysis, strong spin-state dependence in reactivity is well-known in homogeneous catalyst (Schröder et al., 2000) analogs. Thus, it follows that paradigms that work in coordination chemistry might apply to SACs as well. The near-octahedral coordination environment around a metal center produces distinct quantum mechanical spin states (i.e., local metal magnetic moments) that are determined by the ligand-field strength as well as oxidation state and metal identity (Tsuchida, 1938). Different spin states often have distinct reaction barriers (Schröder et al., 2000; Schwarz, 2017) in a paradigm known as two-state reactivity (TSR) (Shaik et al., 1995; Schröder et al., 2000; Schwarz, 2017). TSR was first identified for Fe^+^ ions, where oxidation of H_2_ or CH_4_ to H_2_O or CH_3_OH, respectively, is limited by spin inversion from a steep ground state, high spin (HS) surface to a more reactive but excited state low spin (LS) surface. For cases such as iron-oxo porphyrin systems that have nearly degenerate spin states, different pathways can indeed lead to distinct products (Kamachi and Yoshizawa, 2003; Ji et al., 2015).

In minimal model single-site catalysts, we recently demonstrated (Gani and Kulik, 2018) that bond elongation or compression has an effect similar to modulating ligand field strength, which could also alter ground state spin and reactivity in an interconnected manner. Most Fe(II)/N complexes have near degenerate HS and LS states because nitrogen ligands are of intermediate field strength, but small changes in the N-N separation of bidentate ligands that make up the octahedral complex are known to tune the experimental ground state of the material (Phan et al., 2017). In metal-doped graphene, strain has been predicted to change the ground state spin (Huang et al., 2011). Even changes in orientation of ligands (i.e., one equatorial swapped with one axial) have been experimentally observed to change the favored ground state spin of related molecular complexes (Andris et al., 2016).

The confined nature of metal *d* states and interactions with localized *p* orbitals from organic ligand atoms impart properties to open-shell SACs in a manner more closely resembling the chemical bonding of homogeneous catalysts than bulk metal counterparts. These very features, i.e., quantum size effects (Yang et al., 2013) at an open shell, high-valent metal atom, that make SACs reactive for essential catalytic transformations (Qiao et al., 2011; Yang et al., 2013; Zitolo et al., 2015, 2017; Zhang et al., 2018) also make conventional computational tools used in heterogeneous catalysis (i.e., plane wave, semi-local density functional theory, or DFT) ill-suited to predictive SAC study. Well-localized electrons are disproportionately affected by self-interaction error in approximate DFT (Cohen et al., 2011; Kulik, 2015), leading to an imbalanced treatment of differing spin and oxidation states (Ganzenmüller et al., 2005; Kulik et al., 2006; Droghetti et al., 2012; Ioannidis and Kulik, 2015, 2017; Mortensen and Kepp, 2015; Gani and Kulik, 2017).

Despite evidence of the importance of spin in homogeneous (Abram et al., 2014; Zhu et al., 2016; Schwarz, 2017) and SAC catalysts (Liu et al., 2017), most first-principles studies of SACs (Chu et al., 2015; Ma et al., 2016; Xu et al., 2018) have avoided directly quantifying the role of metal center spin in reactivity, with few exceptions (Impeng et al., 2014; Fong et al., 2018; Sirijaraensre and Limtrakul, 2018). In most studies, the magnetic moment is calculated with a semi-local DFT functional known to produce erroneous magnetic moments (Kulik, 2015; Ioannidis and Kulik, 2017; Janet et al., 2017; Wilbraham et al., 2017) and the magnetization is often allowed to vary along the reaction coordinate (Xu et al., 2018). However, in confined metal centers, spin states are well defined and transitions between spin states occur with low probability because they are quantum mechanically forbidden. Spin state transitions can become kinetically limiting (Shaik et al., 1995; Schröder et al., 2000; Schwarz, 2017), explaining unexpected experimental reactivity (Andris et al., 2016). Within the homogeneous catalysis community (Harvey, 2014; Hernández-Ortega et al., 2015), significant effort has been made to develop tools to assess whether spin crossover is kinetically limiting, but this is not the case in SACs.

Unfortunately, given the importance of spin in predicting reactivity, spin state ordering is highly sensitive to the exchange-correlation functional employed in approximate DFT (Ganzenmüller et al., 2005; Kulik et al., 2006; Droghetti et al., 2012; Ioannidis and Kulik, 2015, 2017; Mortensen and Kepp, 2015). Semi-local (e.g., generalized gradient approximation, GGA) DFT functionals widely employed for their good cost/accuracy balance for many properties consistently stabilize overly-delocalized, covalent states (Autschbach and Srebro, 2014). GGAs thus favor the increased bonding in low-spin over high-spin states (Kulik, 2015; Gani and Kulik, 2017; Ioannidis and Kulik, 2017; Janet et al., 2017; Wilbraham et al., 2017). Hybrid functionals, which incorporate an admixture of HF exchange, are employed in organic chemistry to correct delocalization errors (Kümmel and Kronik, 2008). In transition metal catalysis, the fraction of HF exchange required, as judged by comparison to experiment or accurate correlated wavefunction theory (WFT) reference, is strongly system dependent (Bruschi et al., 2004; Ganzenmüller et al., 2005; Smith et al., 2005; Bowman and Jakubikova, 2012; Droghetti et al., 2012; Ioannidis and Kulik, 2015; Verma et al., 2017).

Thus, in this work, we carry out highly accurate correlated wavefunction theory calculations to develop benchmarks for transition metal complex spin state ordering with ligands that model the environment observed in single atom catalysts. Using these benchmarks, we identify trends in DFT functional performance, and then we evaluate how these observations influence predictions of the stability, reactivity, and ground state identity in models of Fe(II)/N-doped graphene SACs. Finally we provide our conclusions and outlook, including recommendations for computational modeling in this emergent space of single atom catalysis.

## Computational Details

### Octahedral Transition Metal Complexes

Initial structures of octahedral transition metal (TM) complexes with H_2_O, NH_3_, pyridine, and pyrrole ligands were built with the molSimplify toolkit (Ioannidis et al., 2016) with both ligand force-field pre-optimization and trained metal-ligand bond length features enabled. For the hexa-aqua and hexa-ammine complexes, M(II) Ti-Ni and M(III) V-Cu ions were studied, but the pyrrole and pyridine ligands were only studied in complex with Fe(II) or Fe(III). The formal charges assigned to the ligands were neutral in all cases except for pyrrole, which was deprotonated and given a −1 charge. High-spin (HS)-low-spin (LS) states studied in this work were defined as: triplet-singlet for *d*^2^ Ti(II)/V(III) and *d*^8^ Ni(II)/Cu(III), quartet-doublet for *d*^3^ V(II)/Cr(III) and *d*^7^ Co(II)/Ni(III), quintet-singlet for *d*^4^ Cr(II)/Mn(III) and *d*^6^ Fe(II)/Co(III), and sextet-doublet for *d*^5^ Mn(II)/Fe(III). Intermediate-spin (IS) states were also studied: triplet *d*^4^ Cr(II)/Mn(III) or *d*^6^ Fe(II)/Co(III) and quartet *d*^5^ Mn(II)/Fe(III).

### Fe/N-Doped Graphene SAC Finite Models

Two possible Fe(II) coordination environments in finite graphitic SAC models were investigated with DFT, and in both the metal is coordinated by four nitrogen atoms substituted in the graphene structure. In both cases, we employed a hydrogen-atom-terminated graphene flake to avoid increasing computational cost in accordance with prior SAC computational studies that used finite models (Xu et al., 2018). First, a FeN_4_C_10_ compound (chemical formula: C_36_N_4_H_16_Fe) was studied in which all coordinating nitrogen atoms were in six-membered rings (i.e., pyridinic N). This structure is formed by removing two adjacent C atoms from C_42_H_16_ and replacing the four C atoms surrounding the vacancy with N atoms. This active site would correspond to two adjacent point defects in graphene, as has been observed experimentally (Banhart et al., 2011). A second compound, FeN_4_C_12_ (chemical formula: C_40_N_4_H_16_Fe), was also studied in which all coordinating nitrogen atoms were in five-membered rings (i.e., pyrrolic N). This structure was formed by removing two C atoms from a C_46_H_16_ structure, which contains two seven-membered rings in the center surrounded by four five-membered rings. Thus, this structure would require vacancy migration that has also been experimentally observed (Banhart et al., 2011). The two C atoms were removed from where the seven membered rings are joined, and the four inward-facing C atoms that are part of the five-membered rings were replaced with N atoms. All initial coordinates were generated by drawing the 2D structures with ChemDraw and converting the xml structures to 3D coordinates with the molSimplify (Ioannidis et al., 2016) interface to OpenBabel (O'Boyle et al., 2011) followed by force field optimization with the universal force field (Rappé et al., 1992). Singlet, triplet, and quintet spin states were studied, and all simulations had zero net charge.

### Fe/N-Doped Graphene SAC Periodic Models

Periodic analogs to the flake models were studied starting from a 4 × 4 supercell of graphene at its experimental lattice parameter (Trucano and Chen, 1975). A smaller supercell than suggested (i.e., 7 × 7) in previous work (Krasheninnikov et al., 2009) was used for computational efficiency, and future work should focus on the effect of supercell size on dopant properties. The pyridinic (chemical formula: FeN_4_C_18_) SAC model was created following the same vacancy/N-atom replacement approach as in the finite case. For the pyrrolic (chemical formula: FeN_4_C_20_) SAC model, we started from the pyridinic case, inserting C atoms into the five-membered FeN_2_C_2_ ring. Next, we adjusted the adjacent six-membered rings into five-membered rings to create pyrrolic N atoms. In this small supercell, an eight-membered C ring was then formed next to the five membered rings. Neutral systems were studied by spin polarized, fixed magnetization periodic calculations in singlet, triplet, and quintet states.

### Localized Basis Set DFT Calculations

#### Transition Metal Complexes

All LS, IS, and HS complexes were geometry optimized with DFT using the PBE0 (default 25% exchange) global hybrid GGA functional (Adamo and Barone, 1999) with the def2-TZVP basis set (Weigend and Ahlrichs, 2005) in ORCA v.4.0 (Neese, 2018). Singlet states were calculated in a restricted formalism, whereas all remaining calculations were open shell and required level shifting (Saunders and Hillier, 1973) in select cases to aid self-consistent field convergence typically with a value of 1.0 eV but as large as 100.0 eV in one case ([Mn(NH_3_)_6_]^2+^). The optimizations were carried out using the BFGS algorithm in redundant internal coordinates implemented to the default tolerances of 3 × 10^−4^ hartree/bohr for the maximum gradient and 5 × 10^−6^ hartree for the change in self-consistent field (SCF) energy between steps. All calculations at other levels of theory or with differing functional definitions were obtained as single point energies on these optimized geometries. The effect of Hartree-Fock (HF) exchange fraction choice on spin-state energetics within DFT was investigated by altering the fraction in a modified form of the PBE0 global hybrid. The HF exchange fraction was varied from as low as 0% [i.e., a pure PBE GGA (Perdew et al., 1996)] to as high as 100% HF exchange in increments of 10–20%, as indicated in the text, again using the def2-TZVP basis set. In previous work (Gani and Kulik, 2016), we found tuning range-separation parameters in range-corrected hybrids to have a comparable effect on density and energetics of transition metal complexes to global exchange tuning, and therefore we focus on only global exchange tuning in this work.

#### Fe/N-Doped Graphene Flake Models

Geometry optimizations and single-point energy calculations were performed with ORCA v4.0. All DFT methodology was kept the same as for transition metal complexes, including geometry optimizing at PBE0 (25% exchange) and carrying out single points at modified exchange fractions in PBE0 in 10% increments in conjunction with the def2-TZVP basis set, except as noted below. All singlet, triplet, and quintet calculations were carried out in an unrestricted formalism. All calculations employed the resolution of the identity (RI) (Baerends et al., 1973; Whitten, 1973; Dunlap et al., 1979; Eichkorn et al., 1995, 1997; Kendall and Fruchtl, 1997) and the chain-of-sphere (COSX)(Neese et al., 2009) approximations with the auxiliary basis set def2/J (Weigend, 2006) and def2-TZVP/C (Hellweg et al., 2007) for all atoms to accelerate the calculations while introducing marginal errors (Kossmann and Neese, 2009). Molecular structures and orbitals were visualized and plotted with VESTA (Momma and Izumi, 2011).

### Periodic DFT Calculations

All systems were calculated with both the PBE (Perdew et al., 1996) semi-local GGA functional and the HSE06 (Heyd et al., 2003, 2006) local, range-separated GGA hybrid using the plane wave, periodic boundary condition Quantum-ESPRESSO (Paolo et al., 2009) code. Norm-conserving pseudopotentials for C, N, and Fe were generated with OPIUM (Rappe et al., 1990). The wavefunction and charge density cutoffs employed were 50 Ry and 200 Ry, respectively. A Monkhorst-Pack k-point grid of 8 × 8 × 1 was used for efficiency after confirming convergence of total energies with k-point mesh size. Total energies were converged to 0.1 meV and forces were converged to 1 meV/Å. Quantum-ESPRESSO post-processing tools were employed to visualize the spin density and projected density of states. Variable cell relaxation was employed to obtain final lattice parameters with PBE GGA for the pyridinic (result: 8.34 Å × 7.55 Å) and pyrrolic (result: 8.35 Å) models. A vacuum between each SAC layer of 10 Å was included along with a dipole correction to limit periodic image effects (Bengtsson, 1999). The HSE06 calculations were obtained as single point energies applied to these structures.

### Correlated WFT

Complete active-space second-order perturbation theory (CASPT2) (Andersson et al., 1992) calculations were performed with OpenMolcas^1^ (Aquilante et al., 2016) on M(II)/M(III) hexa-aqua and hexa-ammine octahedral complexes. Calculations were carried out with two active space definitions: the standard active space and an extended active space. For the standard active space, we followed literature recommendations for TM complexes (Pierloot, 2003; Veryazov et al., 2011) to include five orbitals with TM 3*d* character, two σ bonding orbitals describing covalent metal-ligand bonding, and five double-shell *d* orbitals for mid-row and later transition metals [i.e., Mn(II/III) and later]. For n 3*d* electrons, the standard active space is (n + 4, 7) (i.e., for Sc-Cr) or (n + 4, 12) (i.e., for Mn-Cu). In the extended active space, we followed additional literature recommendations (Wilbraham et al., 2017) to include the metal 3*s* orbital and an unoccupied counterpart, giving an active space of (n + 6, 9) (i.e., for Sc-Cr) or (n + 6, 14) (i.e., for Mn-Ni). Relativistic atomic natural orbital (ANO-rcc) basis sets (Roos et al., 2004, 2005) contracted to [7s6p5d3f2g1h] for the metal center, [4s3p2d1f] for O and N, and [3s1p] for H were used together with the scalar relativistic Douglas-Kroll Hamiltonian (Douglas and Kroll, 1974; Hess, 1986). The 10 core orbitals were frozen in all calculations. An imaginary level shift (Forsberg and Malmqvist, 1997) of 0.1 was used, and a zeroth-order Hamiltonian empirical correction, i.e., the IPEA shift (Ghigo et al., 2004), was set as 0.5 a.u. or varied as described in the main text to identify the effect on spin state energetics. For difficult to converge complete active space self-consistent field iterations, a level shift was applied to the Hamiltonian with shift value 1.0.

## Results and Discussion

### Spin State Ordering in Model Complexes

#### Correlated WFT Results

We first conducted correlated wavefunction theory (WFT) calculations to generate reference spin-splitting energies of model first row octahedral transition metal complexes. Our focus is on weak field hexa-aqua and hexa-ammine complexes that are small (i.e., 19–25 atoms in size) and of comparable ligand field strength to the coordination environment in SACs. Although CASPT2 is often the method of choice for predicting spin state energetics for molecules that are either too large or multireference in character to be comfortably treated with CCSD(T) (Pierloot et al., 2017), a number of calculation parameters can strongly influence the CASPT2 predictions. Specifically, spin state energetics can be influenced by the active space choice and the formulation of the zeroth-order Hamiltonian, i.e., the value of the IPEA shift (Ghigo et al., 2004). Here, we investigate the effects of both of these factors and then select reference results for DFT calculations.

**Figure 2:**
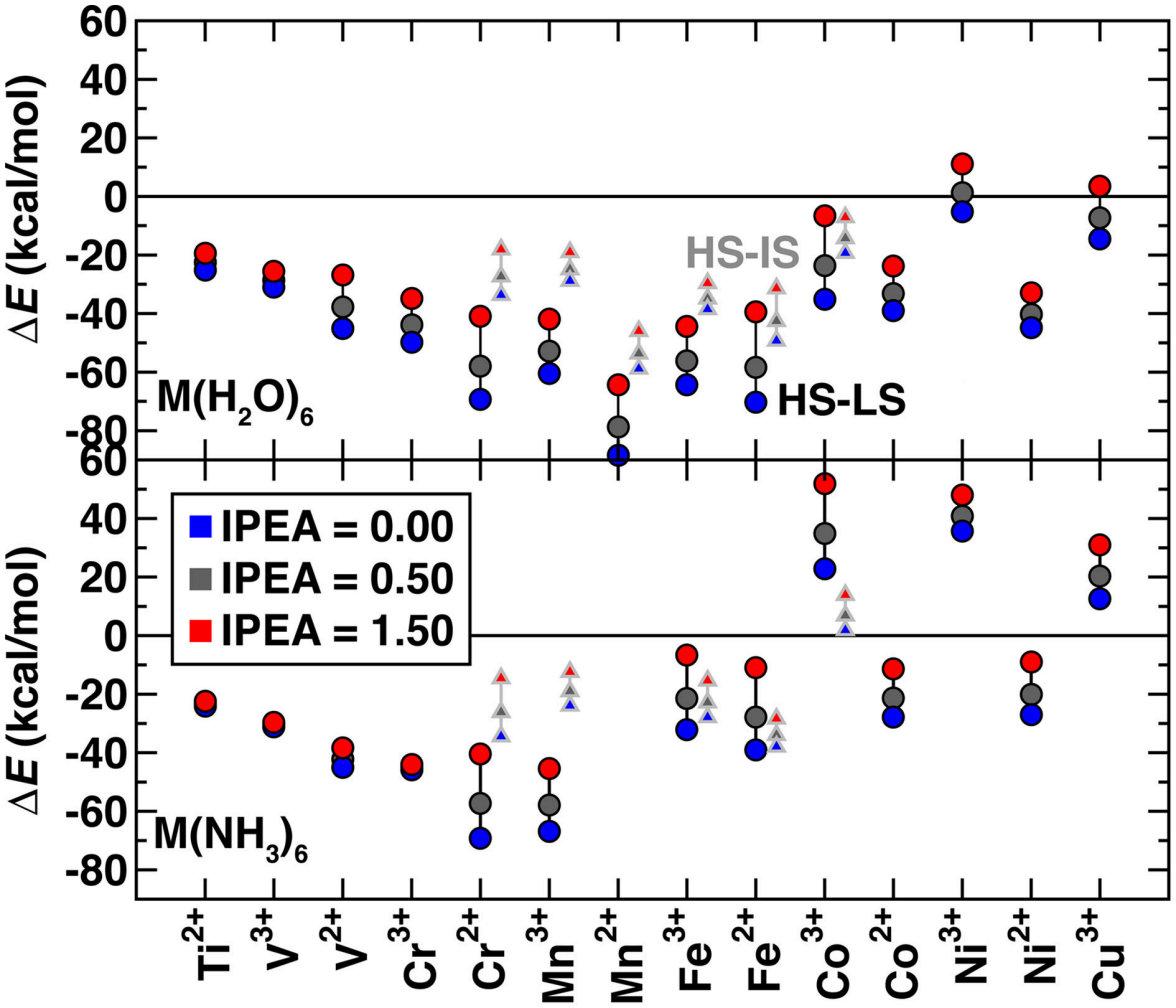
CASPT2 spin splitting energetics in kcal/mol for Δ*E*_H−L_ (circles) and Δ*E*_H−I_ (triangles), as indicated in inset, of hexa-aqua (top) and hexa-ammine (bottom) transition metal complexes. Results are shown for 3 IPEA shifts: 0.00 (blue symbols), 0.50 (dark gray symbols), and 1.50 (red symbols) a.u.. Both M(II) and M(III) complexes are shown sorted by the number of 3*d* electrons, from Ti^2+^ to Cu^3+^.

Several studies (Kepenekian et al., 2009; Lawson Daku et al., 2012; Vela et al., 2016; Pierloot et al., 2017) have shown that the standard IPEA shift of 0.25 a.u. in CASPT2 overstabilizes high spin states. However, there is no universal agreement about the best solution to this problem. Some (Kepenekian et al., 2009; Lawson Daku et al., 2012; Vela et al., 2016) have recommended increasing the IPEA shift to 0.5–0.7 a.u. based on comparison with experimental or MRCI results, whereas others (Pierloot et al., 2017) recommend the standard IPEA value because increased IPEA can reduce the high spin bias but only at the expense of deteriorating the CASPT2 description of valence correlation. To understand the effect of IPEA shift, we obtained CASPT2 spin-splitting energies with IPEA shifts of 0.0, 0.50, and 1.5 a.u. and standard active spaces (Figure 2 and see Computational Details). For all complexes, increasing IPEA shifts the high-spin/low-spin splitting toward more positive values, reducing high spin stabilization. The range of energetics calculated with different IPEA values for each complex provides a measure of IPEA sensitivity of each transition metal complex's spin-splitting energy (Figure 2). The IPEA sensitivity is consistently largest for cases where the high spin state has four more unpaired electrons than the low spin state (i.e., Cr^2+^ through Co^3+^).

To focus only on spin state definitions in which the high spin state has two more unpaired electrons, we compare the high spin/low spin splitting, Δ*E*_H−L_, for early and late TMs with the high spin/intermediate spin splitting, Δ*E*_H−I_, for mid-row TMs. For these two electron differences, we observe similar sensitivities, with a ca. 10–20 kcal/mol positive shift when the IPEA is changed from 0.0 to 1.5 a.u. (Figure 2). This observation excludes only very early or late TM complexes that have smaller sensitivities. In comparison, the 4 electron-difference cases have higher sensitivities of around 30 kcal/mol or more (Figure 2). Overall, most early- and mid-row complexes remain high spin regardless of the IPEA shift (i.e., below the zero axis in Figure 2). The smaller energetic differences between states in later TM complexes [e.g., hexa-aqua Ni(III)] mean that the IPEA shift can change the ground state from high spin to low spin for very large IPEA shifts. For subsequent calculations, we selected the 0.5 a.u. IPEA shift but note that variation across the commonly employed range of 0.0–0.5 a.u. can shift the predicted Δ*E*_H−L_ (Δ*E*_H−I_) spin-splitting for mid-row [e.g., Fe(II/III)] complexes by around 10 (3) kcal/mol (Figure 2).

We next investigated the effect of use of a standard active space vs. a more extended active space in the CASPT2 calculations of Δ*E*_H−L_ and Δ*E*_H−I_ using the 0.5 a.u. IPEA shift (Supplementary Table 1). For most complexes, the energetic difference due to active space change is on the order of a few kcal/mol, which suggests that the calculation is converged with respect to active space size, as motivated in previous work (Wilbraham et al., 2017). The major outlier identified is [Mn(NH_3_)_6_]^2+^, which is strongly low-spin in the standard active space but becomes high-spin like hexa-aqua Mn(II) with the extended active space, exhibiting greater active space dependence than had been observed in Mn(II) porphyrins (Yang et al., 2016) (Supplementary Table 1). This discrepancy is likely caused by orbital rotation of some active orbitals into the inactive metal 3*s*/4*s* orbitals, as suggested in recent work (Radoń and Drabik, 2018) on aqua complexes. After removing this outlier, the mean absolute difference between the standard and extended active space results for all Δ*E*_H−L_ and Δ*E*_H−I_ combinations is 3.3 kcal/mol for the hexa-aqua and 5.9 kcal/mol for the hexa-ammine complexes. The mean signed error is near zero for the hexa-aqua cases, and weakly negative (ca. −3 kcal/mol) for the hexa-ammines (Supplementary Table 1). Generally, discrepancies are smallest for the hexa-aqua complexes throughout and especially small for the early or late TMs (e.g., Ti^2+^-V^2+^ and Ni^3+^-Cu^3+^), typically as little as 0–2 kcal/mol (Supplementary Table 1). In mid-row cases, there is no universal preference as to whether Δ*E*_H−L_ or Δ*E*_H−I_ has more active space dependence.

Given the small size of the studied octahedral complexes, we selected the extended active space calculations as reference values for comparison to DFT. Use of the larger active space changed some ground state spin assignments. When calculated with the extended active space, almost all hexa-aqua complexes are high spin, excluding only weakly low spin [Ni(H_2_O)_6_]^3+^ with a Δ*E*_H−L_ of 0.49 kcal/mol. The slightly stronger ligand field in the hexa-ammine complexes produces some additional LS late-TM complexes [e.g., Co(III) and Cu(III)] along with the analogous Ni(III) complex. Examining isoelectronic metals generally reveals that the later, more oxidized metal has only a weak high-spin-stabilizing effect that is smaller than the ligand-field effect in cases where the two metals converge to similar electronic states (Figure 2 and Supplementary Table 1).

#### DFT Functional Performance

Despite the high accuracy of CASPT2 for treating spin state energetics in TM complexes, the high computational cost and sensitivity to active space definition and parameters limit its application on SACs. We thus sought to identify the extent to which DFT functionals can be selected or tuned to reproduce the spin-splitting energetics obtained with CASPT2. We focused on the exchange fraction within the global hybrid PBE0 (Adamo and Barone, 1999), motivated by previous observations of comparable behavior in tuning range-separated hybrids (Gani and Kulik, 2016), global hybrids with a different correlation functional (Ioannidis and Kulik, 2015), or those that incorporate meta-GGA exchange (Ioannidis and Kulik, 2017).

From all complexes studied with CASPT2, we narrowed our focus to those containing nominally 3–7 3*d* electrons (i.e., V^2+^ to Ni^3+^) that are most likely to be good candidates for understanding spin-state dependent single atom catalysis. We determined the effect of varying PBE0 exchange fraction on both Δ*E*_H−L_ and Δ*E*_H−I_ for the relevant subset (i.e., 4–6 3*d* electrons). In accordance with prior work (Droghetti et al., 2012; Ioannidis and Kulik, 2015), we anticipated the sensitivity of these quantities to exchange fraction and the optimal exchange fraction to minimize error with respect to CASPT2 to be chemistry dependent. However, we aimed to identify if these trends can be readily rationalized (Gani and Kulik, 2017) or learned (Janet and Kulik, 2017) for use in SAC modeling.

As expected (Droghetti et al., 2012; Ioannidis and Kulik, 2015; Zhao and Kulik, 2018), spin-state energetics vary linearly with exchange fraction over a wide range (i.e., 0–50%) and high-spin states are stabilized with increasing exchange fraction for all transition metal complexes studied (Supplementary Tables 2–3). We quantified the exchange sensitivity of the spin-splitting energetics (Ioannidis and Kulik, 2015) by an approximate linear fit:

(1) $$\begin{array}{r} \frac{\partial \Delta E_{\text{H-L/I}}}{\partial a_{\text{HF}}}\approx \frac{\Delta \Delta E_{\text{H-L/I}}}{\Delta a_{\text{HF}}} \end{array}$$

**Figure 3:**
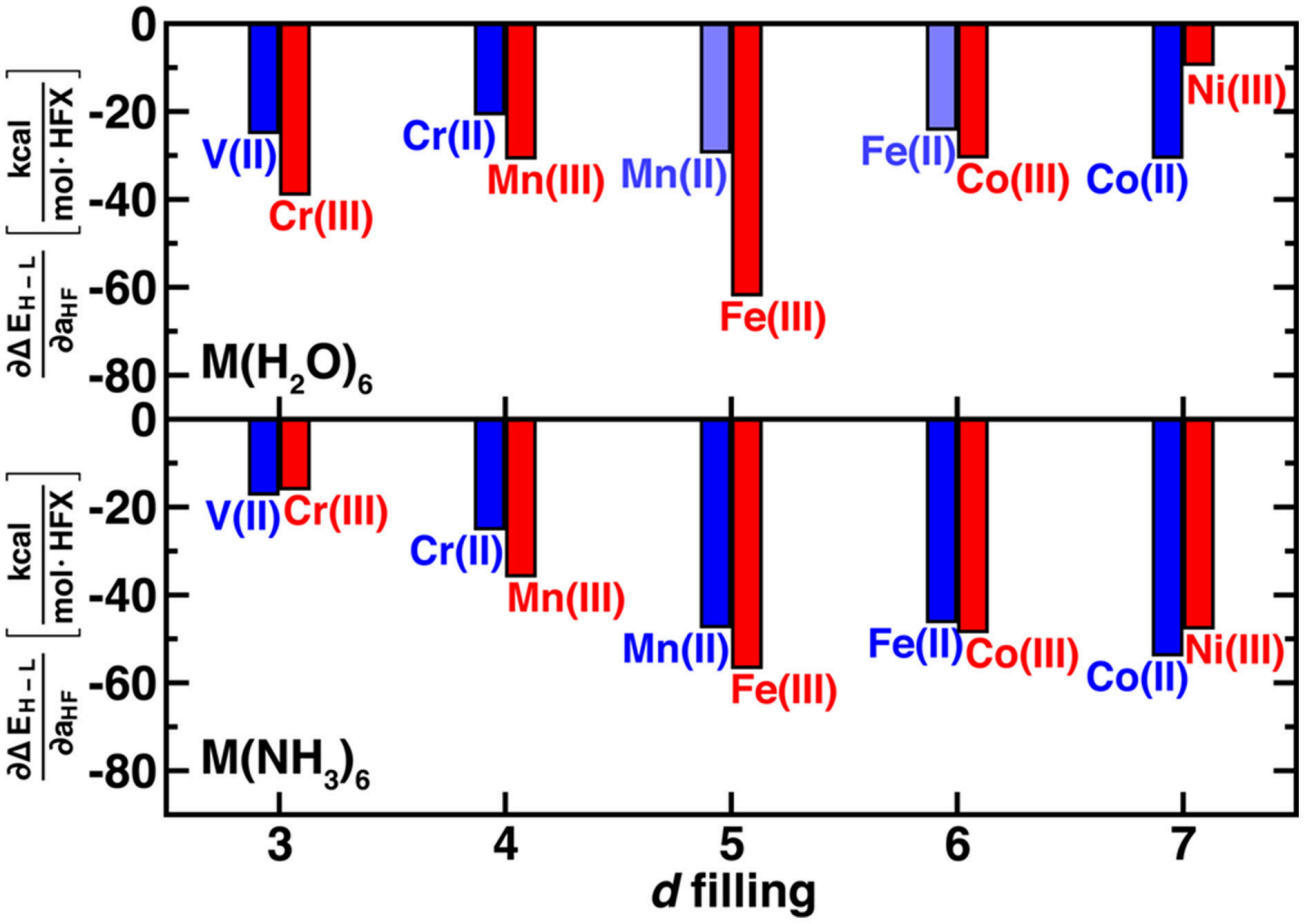
Sensitivity of spin-state splitting with respect to HF exchange (i.e., ∂Δ*E*_H−L_/∂*a*_HF_, in kcal/mol^.^HFX) for hexa-aqua (top) and hexa-ammine (bottom) transition metal complexes. Both M(II) and M(III) complexes are shown grouped by their nominal *d* filling from 3 to 7 3*d* electrons for V(II) to Ni(III). For the 4, 5, and 6 *d*-electron cases, the energy gap corresponds to high-spin/intermediate-spin rather than high-spin/low-spin. Shaded bars indicate that spin contamination could not be eliminated for both spin states and sensitivity may not be reliable.

We use the unit notation HFX corresponding to the variation from 0 to 100% exchange. To maximize correspondence in quantities compared, we evaluate exchange sensitivity of all spin states that differ by two paired electrons: Δ*E*_H−L_ for V(II)/Cr(III) and Co(II)/Ni(III) and Δ*E*_H−I_ for the remaining complexes (Δ*E*_H−L_ values are also tabulated in Supplementary Table 3). The exchange sensitivity of two-electron-difference spin-state ordering for [M(H_2_O)_6_]^2+^ complexes is relatively invariant to 3*d* filling (i.e., varying only 2–4 kcal/(mol·HFX), see Figure 3). Conversely, hexa-ammine Mn(II), Fe(II), and Co(II) complexes have increased spin-splitting exchange sensitivity over the earlier TM complexes (Figure 3). For both ligand fields, the M(III) complexes are even more varied, with the least exchange sensitivity being observed in either late or early transition metal complexes (Figure 3). These observations are consistent with the fact that these complexes should have the least difference in electron delocalization between the two spin states, reducing exchange sensitivity (Gani and Kulik, 2017). Although M(III) complexes are more variable, isoelectronic +2/+3 complexes do have somewhat comparable exchange sensitivity (Figure 3). In comparing Δ*E*_H−L_ for all complexes, exchange sensitivity is universally higher for these mid-row cases due to the enhanced sensitivity of the four-electron difference energetics (e.g., [M(H_2_O)_6_]^2+^: −57 kcal/(mol·HFX) for Mn vs. −25 kcal/(mol·HFX) for V, see Supplementary Figure 1).

**Figure 4:**
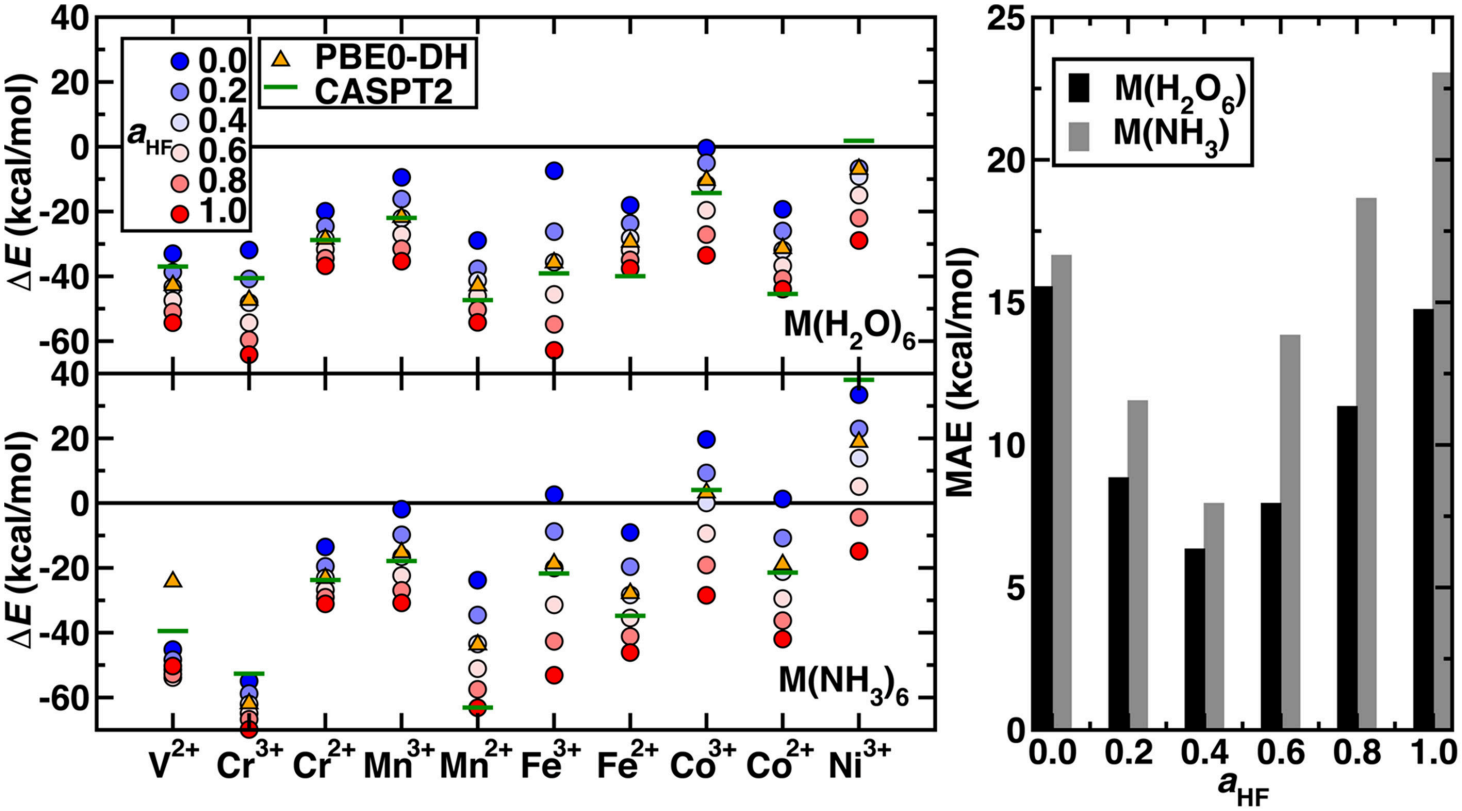
**(Left)** Spin-splitting energetics (in kcal/mol) corresponding to Δ*E*_H−I_ for Cr^2+^-Co^3+^ and Δ*E*_H−L_ for all other cases shown for hexa-aqua **(Top)** and hexa-ammine **(Bottom)** complexes. Modified PBE0 GGA hybrid results are shown as circles. The exchange fraction, *a*_HF_, is colored from blue for 0.0 (pure GGA) to red for 1.0 (full HF exchange), according to the inset legend. Reference CASPT2 results with the extended active space and IPEA shift of 0.5 a.u. are shown as green horizontal lines. The PBE0-DH results are shown as orange triangles. **(Right)** MAE (in kcal/mol) at several exchange fractions for the hexa-aqua and hexa-ammine complexes indicated at left.

The remaining question is whether the exchange fraction can be appropriately tuned within a modified form of PBE0 to obtain Δ*E*_H−L_ and Δ*E*_H−I_ values that match CASPT2 results. Comparing the range of spin-splitting energies obtained from 0 to 100% exchange to the CASPT2 extended active space values generally reveals that high exchange fractions (c.a. 40%) are required to reproduce CASPT2 results (Figure 4 and Δ*E*_H−L_-only results shown in Supplementary Figure 2). With the exception of Ni(III) or V(II)/Cr(III) hexa-ammines, pure PBE GGA Δ*E* values are much more positive (i.e., low-spin biased) than the WFT results (Figure 4). Increasing exchange thus in most cases improves agreement with WFT, but the optimal exchange fraction for reproducing the CASPT2 result varies significantly for the different complexes (Figure 4). For five of the cases [e.g., midrow Mn(II)(H_2_O)_6_, Fe(II)(NH_3_)_6_, and Co(II)(H_2_O)_6_], the optimal exchange fraction is larger than 0.4, whereas the majority of the remaining complexes would require exchange fractions of 0.0-0.4 to recover the CASPT2 value (Figure 4). Only three complexes [i.e., V(II)(H_2_O)_6_, Cr(III)(H_2_O)_6_, and Ni(III)(H_2_O)_6_] have an optimal exchange fraction corresponding to typically applied (Ioannidis and Kulik, 2015) values of around 10–30%.

Considering overall performance on the 20 TM complexes, incorporating any exact exchange reduces the mean absolute error (MAE) of spin-splitting energies with respect to WFT. From PBE to 20% to 40% exchange, the MAE decreases monotonically from 17 to 12 to 8 kcal/mol for hexa-ammines and comparably (i.e., 16 to 9 to 6 kcal/mol) for hexa-aquas (Figure 4). Increasing HF exchange higher than 40% increases the MAE again for both ligand fields. For these two weak ligand fields, the optimal exchange fraction is more metal dependent than ligand field dependent, producing comparable optimal exchange values for fixed metal and oxidation state (Figure 4). Recent work (Wilbraham et al., 2018) has suggested double hybrids (DH, i.e., with MP2 long-range correlation) could improve predictions of spin-state ordering. We selected the PBE0-DH, which contains 50% global HF exchange for comparison to GGA global hybrid results (Figure 4). The PBE0-DH results are comparable to those obtained with a modified PBE0 global hybrid GGA with 40% exchange (i.e., MAE of 8 kcal/mol for hexa-ammine complexes and 6 kcal/mol for hexa-aqua complexes, see Supplementary Table 4). Given the higher computational cost and scaling of the double hybrids, tuned GGA hybrids would remain a preferable choice for modeling larger systems.

#### Comparison of Nitrogen-Containing Ligands

**Figure 5:**
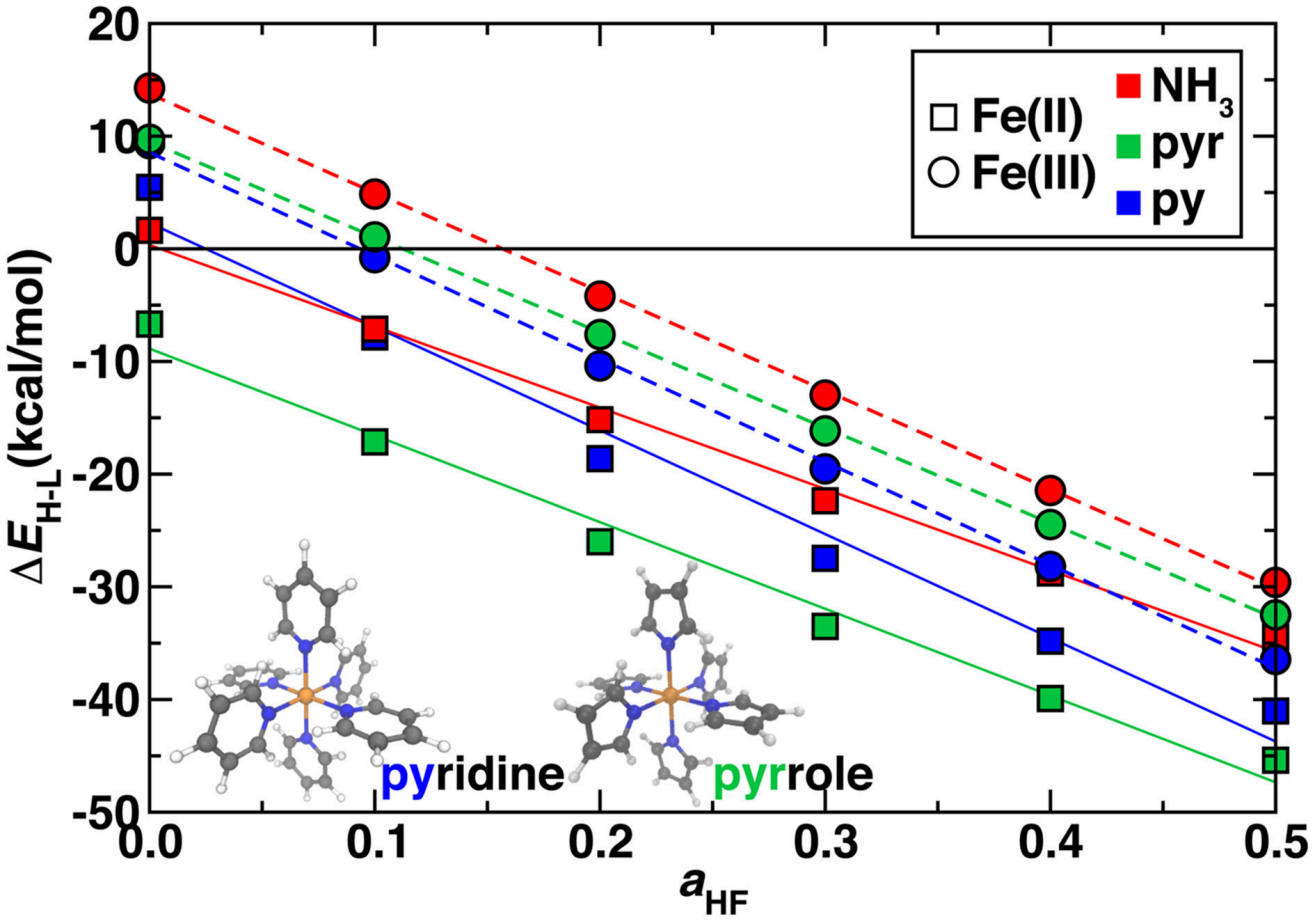
Sensitivity to HF exchange fraction (*a*_HF_) of high-spin/low-spin splitting (Δ*E*_H−L_, in kcal/mol) for Fe(II) (squares) and Fe(III) (circles) homoleptic octahedral transition metal complexes with NH_3_ (red symbols), pyridine (blue symbols), and pyrrole (green symbols) ligands. The structures of pyridine and pyrrole compounds are shown in inset in ball and stick (gray carbon, blue nitrogen, white hydrogen, and orange for iron). A zero axis is shown that indicates change in favored ground state spin.

So far, we have studied model complexes and confirmed the importance of incorporating exact exchange in DFT functionals to reproduce correlated WFT reference spin-splitting energetics. We next considered the transferability of exchange sensitivity of DFT spin-splitting energetics to octahedral transition metal complexes that contain coordination environments similar to Fe/N-doped graphene SAC models. The two ligands we used to represent these environments were pyridine (py) in which the coordinating nitrogen is in a six membered carbon-containing ring and pyrrole (pyr) in which the coordinating nitrogen is in a five membered carbon-containing ring (Figure 5 inset and see Computational Details). With a pure PBE GGA, all complexes except for hexa-pyr Fe(II) are low spin, whereas exchange fractions above ~15% instead result in all ground states being assigned as high spin (Figure 5). The trend with exchange is again linear as in the hexa-ammine complexes, although the linearity is slightly reduced for Fe(II) vs. Fe(III) complexes (Figure 5).

The Δ*E*_H−L_ values are within ~7 kcal/mol for all complexes of the same oxidation state across the full range of exchange due to the relatively similar ligand field strengths of the N-containing ligands and comparable metal-ligand bond lengths (Supplementary Table 5). However, the hexa-ammines are generally the most low-spin-favoring, whereas the SAC-like nitrogen complexes have a slightly increased high-spin bias. In analogy to ligand field arguments for Δ*E*_H−L_, exchange sensitivities, $\frac{\partial \Delta E_{\text{H}-\text{L}}}{\partial a_{\text{HF}}}$, of spin splitting are also comparable for ammonia, pyridine and pyrrole complexes (Figure 5). For $\frac{\partial \Delta E_{\text{H}-\text{L}}}{\partial a_{\text{HF}}}$, NH_3_ and pyrrole are very similar in both Fe(II) [−72 and −77 kcal/(mol·HFX)] and Fe(III) [−88 and −85 kcal/(mol·HFX)], whereas pyridine has slightly larger slope of −92 kcal/(mol·HFX) in both oxidation states. These observations are consistent with the previously observed greater sensitivity to the ligand identity than to oxidation state for Fe(II)/Fe(III) (Ioannidis and Kulik, 2015). Due to the similar spin-splitting energetics and sensitivities to exchange, we expect that our observations on hexa-ammine complexes are applicable to pyridinic and pyrrolic nitrogen-containing complexes and materials as well. Thus, for larger SAC models, we recommend either typical exchange fractions for qualitative ground state spin assignment (i.e., high spin) or higher exchange fractions (ca. 40–50%) that were needed in the hexa-ammine cases to reproduce WFT results quantitatively.

### Graphene Flake Models of SACs

**Figure 6:**
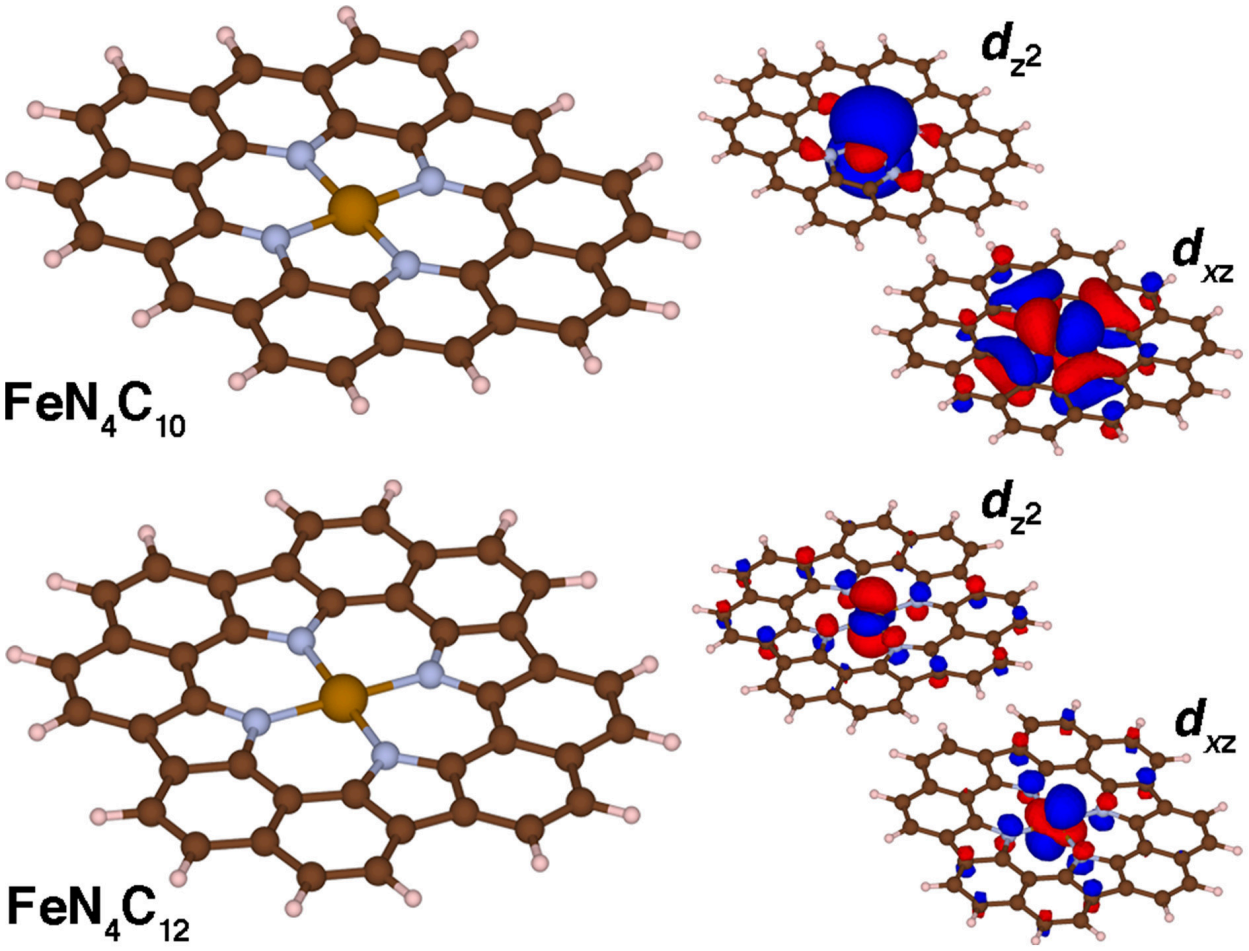
Molecular structures (left) and singly occupied *d*_*xz*_ and *d*_*z*_^2^ spin-up molecular orbitals (right) for FeN_4_C_10_ and FeN_4_C_12_ graphene flake SAC models in the triplet state. The positive and negative phases of the wavefunction are shown in red and blue, respectively. An isosurface of 0.01 *e*/Bohr^3^ was used for the orbitals of FeN_4_C_10_ and of 0.03 *e*/Bohr^3^ for those of FeN_4_C_12_ for clarity. All structures are shown in ball and stick representation with carbon in brown, hydrogen in white, nitrogen in light blue, and iron in orange.

Fe/N-doped graphitic SACs are expected on the basis of experimental spectroscopic characterization (Zitolo et al., 2015; Chen et al., 2017; Liu et al., 2017) to consist of Fe metal centers coordinated by pyridinic or pyrrolic nitrogen atoms. These experimental observations come from a combination of aberration corrected scanning tunneling electron microscopy to confirm well-isolated metal sites as well as numerous spectroscopic techniques (e.g., X-ray absorption spectroscopy) to confirm the metal coordination (Zitolo et al., 2015; Chen et al., 2017; Liu et al., 2017). Although the most reactive SAC active site remains an open question, we consider in this work two limits in finite graphene flake SAC models that contain either four pyridinic (FeN_4_C_10_) or pyrrolic (FeN_4_C_12_) N atoms (Figure 6). Due to the rigidity of the graphene flakes, singlet Fe(II) pyridinic (py) and pyrrolic (pyr) Fe-N bond lengths are shorter than the corresponding py or pyr octahedral complexes (py: 1.90 vs. 2.08 Å or pyr: 1.97 Å vs. 2.11 Å, see Supplementary Tables 5–6). This rigidity in the SAC models without any displacement of iron from the plane that has been observed in porphyrins (Sahoo et al., 2015) also leads to average Fe-N distances being invariant to spin state. There is a marginal (ca 0.01 Å) increase from singlet to quintet spin states for the FeN_4_C_10_ SAC in comparison to large (ca. 0.16–0.20 Å) bond length increases from singlet to quintet in the Fe(II)(py)_6_ complex (Supplementary Tables 5–6). The Fe-N distances are shorter in pyridinic FeN_4_C_10_ than in pyrrolic FeN_4_C_12_ (i.e., 1.90 Å vs. 1.96 Å) due to smaller N-N separations (FeN_4_C_10_: 2.61 Å and 2.75 Å, FeN_4_C_12_: 2.75 Å). Despite this difference in N-N separation, which has previously been noted to influence experimental spin state ordering (Phan et al., 2017), the PBE0 ground state spin is triplet in both models (Supplementary Table 7).

The two singly occupied orbitals in both triplet SAC models correspond to the *d*_xz_ and *d*_*z*_^2^ orbitals consistent with expectations for square-planar coordinated triplet Fe(II) (Figure 6). Small differences are observed in the orbital character due to the lower symmetry for the pyridinic FeN_4_C_10_ flake: in this case, *d*_*xz*_ and *d*_*yz*_ degeneracy is broken (Figure 6). The longer N-N separation along the *x*-axis leads to a pure *d*_*xz*_ orbital vs. *d*_*xz*_ and *d*_*yz*_ mixing for the case of pyrrolic FeN_4_C_12_ (Figure 6). In both cases, weak coupling is observed between the metal-centered orbitals and *p*-orbitals of both the N and C atoms in the graphene flake (Figure 6).

For both SAC models at the PBE0 level of theory, singlet and quintet states reside ~4–6 kcal/mol and 12 kcal/mol above the triplet ground state, respectively, and thus singlet-quintet Δ*E*_H−L_ is around +6–8 kcal/mol (Supplementary Table 7). These observations contrast with the octahedral models: Δ*E*_H−L_ for Fe(II)(py)_6_ is −15 kcal/mol and is −30 kcal/mol for Fe(II)(pyr)_6_ (Supplementary Table 4). These differences can be traced to several factors, including the coordination number (4 vs. 6) in the models as well as rigidity of the graphene flakes that compress Fe-N bonds to values more commensurate with equilibrium low-spin geometries. Finally, examining the electronic structure of the SAC models reveals distribution of spin not just on the metal but also on the flake in the high spin states, particularly for the pyrrolic FeN_4_C_12_ models (Supplementary Tables 8, 9). Even in the triplet ground state this is apparent with a magnetic moment of 2.2 μ_B_, close to that expected (i.e., 2 μ_B_) for FeN_4_C_10_, but with a larger 2.7 μ_B_ on Fe for FeN_4_C_12_ (Supplementary Tables 8, 9). The singlet FeN_4_C_12_ is also open shell with a 1.4–2.0 μ_B_ magnetic moment on Fe (Supplementary Table 9). In contrast with the molecular complexes, the quintets are particularly poorly described by a localized metal spin, with a reduced Fe moment vs. the triplet state of around 2.4 μ_B_ on Fe for FeN_4_C_12_ and a comparable one of 2.2 μ_B_ on Fe for FeN_4_C_10_. Although spin contamination can be expected with increasing HF exchange fraction, comparison of these moments across 0–50% exchange does not ever produce a pure 4 μ_B_ moment on Fe for the quintet FeN_4_C_12_ (Supplementary Table 9). This observation could be due to low-lying unoccupied states on graphene that are populated instead of the metal states, especially in these models and at this level of theory, as is known to occur in porphyrins as well (Fujii, 2002).

**Figure 7:**
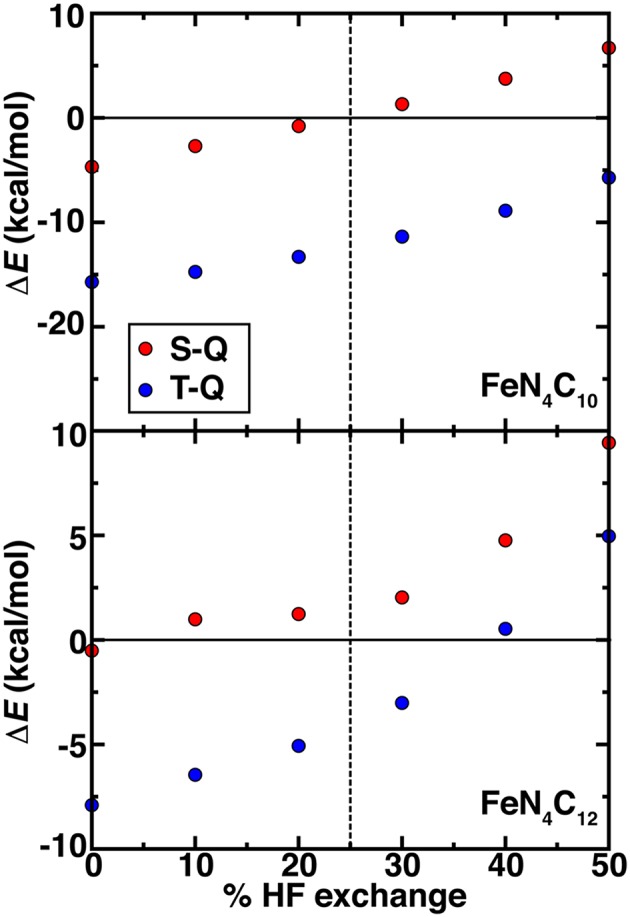
Spin-splitting energetics (in kcal/mol) for singlet-quintet (S-Q, red circles) or triplet-quintet (T-Q, blue circles) spin states vs. % HF exchange for pyridinic (FeN_4_C_10_, top) and pyrrolic (FeN_4_C_12_, bottom) SAC models. The 25% exchange in standard PBE0 is indicated as a vertical dashed line.

Beyond PBE0 (25%) results on the graphene SAC models, we considered properties over a range that spans from typical values in periodic catalysis modeling (i.e., 0%) to larger values (40%) motivated by our octahedral complex studies (see section Spin State Ordering in Model Complexes). Over this 0–40% range of exchange fractions, singlet and triplet states become destabilized with respect to high-spin quintet states (Figure 7). The reduced dependence of spin-state ordering observed here on exchange fraction in comparison to the octahedral complexes is due to differences in coordination number and rigidity of the SAC models. Intermediate Fe spin states were observed experimentally (Liu et al., 2017) for N-doped graphitic SACs using Mössbauer spectroscopy. Although high HF exchange fractions favoring quintet states for both SAC models would suggest inconsistencies with experiment, it is important to recall that the magnetic moments of the Fe metal are intermediate in both triplet and quintet states. Therefore, high HF exchange fractions are in fact stabilizing the simultaneous presence of spin on the graphene coupled to an intermediate Fe center (Supplementary Tables 8, 9). In both FeN_4_C_12_ at low exchange fractions and FeN_4_C_10_ over a larger range of 0-100% exchange, higher order than typically linear sensitivities are observed to % exchange (Supplementary Figure 3). For FeN_4_C_12_, the anti-ferromagnetically coupled metal spin on Fe varies significantly (i.e., 0.6 μ_B_) and discontinuously, leading to less smooth energetic variations (Figure 7). For the FeN_4_C_10_ model where spin states are more well defined, the variations are instead linear over the expected range of HF exchange (Droghetti et al., 2012; Ioannidis and Kulik, 2015; Zhao and Kulik, 2018).

**Figure 8:**
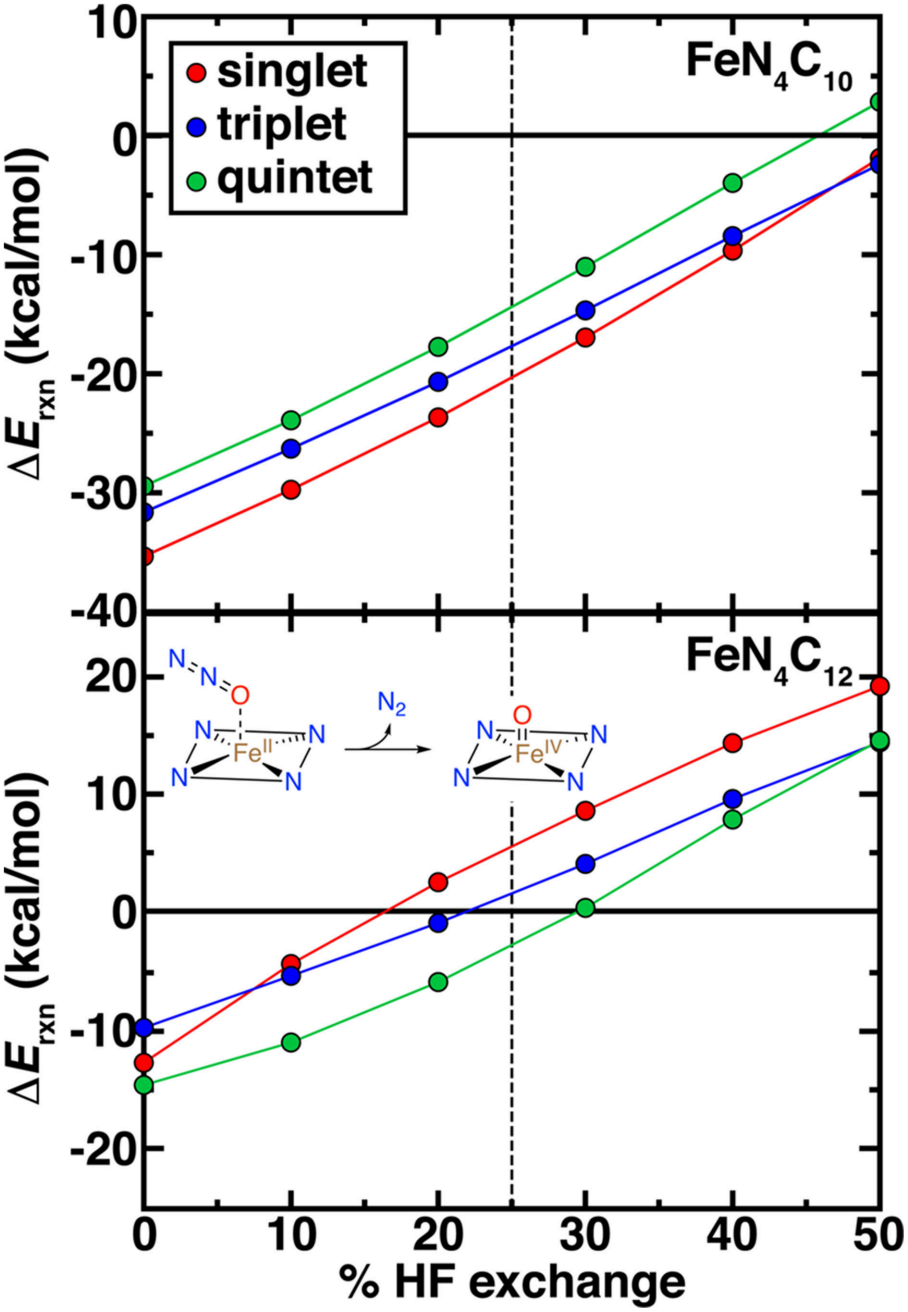
Reaction energetics (Δ*E*_rxn_, in kcal/mol) for oxo formation from N_2_O oxidant vs. % HF exchange for pyridinic (FeN_4_C_10_, top) and pyrrolic (FeN_4_C_12_, bottom) SAC models. The reaction is shown in inset. In each case, singlet (red circles), triplet (blue circles), and quintet (green circles) oxo formation energies are shown. The 25% exchange in standard PBE0 is indicated as a vertical dashed line.

High-valent Fe(IV) = O intermediates are expected to be essential for catalytic transformations at N-doped graphitic SACs (Liu et al., 2017). Thus, we examined the spin-state-, model-, and exchange-fraction-dependence of reaction energetics for Fe(IV) = O formation. Here, we employed N_2_O as a model oxidant, but results are comparable when assuming the oxygen atom comes from triplet O_2_ (Figure 8 and Supplementary Figures 4–6). Overall, pyridinic SACs form more stable oxo species across the range of HF exchange and spin states than pyrrolic SACs (Figure 8). Although activation energies would be needed to make firmer statements about relative active site model reactivity, the endothermic reaction energies for the intermediate spin FeN_4_C_12_ above 20% exchange (ca. +10 kcal/mol at 40% exchange) suggest that the pyrrolic model could potentially be unreactive with N_2_O oxidant (Figure 8). Regardless of spin state or model, increasing exchange fraction makes formation of oxo intermediates less favorable due to the penalty for delocalization (Gani and Kulik, 2017).

Determination of ground state spin from DFT of the pristine Fe(II)/N SAC model and the Fe(IV) = O intermediate should be carried out with caution, noting that spin (ca. 0.5–1.0 μ_B_) arises on the O atom in triplet and quintet states of both Fe(IV) = O SAC models but not in the singlet states (Supplementary Tables 8, 9). Although spin on the oxo species can be expected and even linked to catalytic efficiency (Liu et al., 2009; Quesne et al., 2014), these states cannot readily be described within a single Kohn-Sham determinant in DFT (Koch and Holthausen, 2015). Overall, spin state ordering of the bare Fe(II) SAC is largely preserved in the Fe(IV) = O intermediate, with singlet and triplet Fe(IV) = O states being weakly stabilized by around 3–5 kcal/mol with respect to the bare Fe(II) case for the pyridinic SACs (Supplementary Figure 4). In the pyrrolic case, the opposite occurs, potentially due to the loss of spin on the ring in triplet pyrrolic Fe(IV) = O (Supplementary Table 9 and Figure 4). Nevertheless, further investigation of kinetic barriers is merited in future work, as close spin state ordering of both the Fe(IV) = O and pristine Fe(II) intermediates combined with comparable differences of around 5–10 kcal/mol between reaction energetics in each spin state could give rise to spin-state dependent reactivity with distinct product formation (Kamachi and Yoshizawa, 2003; Ji et al., 2015). Finally, it is noteworthy that at 0% exchange (i.e., pure PBE), singlet FeN_4_C_12_ is predicted to produce a slightly more stable Fe(IV) = O than the triplet, the same ordering that is observed for FeN_4_C_10_ albeit at −10 to −15 kcal/mol in the former case vs. −30 to −35 kcal/mol in the latter case (Figure 8). At the 40% exchange motivated by our careful CASPT2 characterization of Fe-N bonds (see section Comparison of Nitrogen-Containing Ligands), or even at the 25% exchange fraction motivated in stronger ligand field cases (Ioannidis and Kulik, 2017), conclusions are different. Namely, at these higher exchange fractions: (i) the triplet oxo is more stable for pyrrolic SACs than the singlet, whereas the ordering remains the same for the pyridinic case, and (ii) neither form exothermically for the pyrrolic case at these exchange fractions.

### Periodic Modeling of SACs

**Figure 9:**
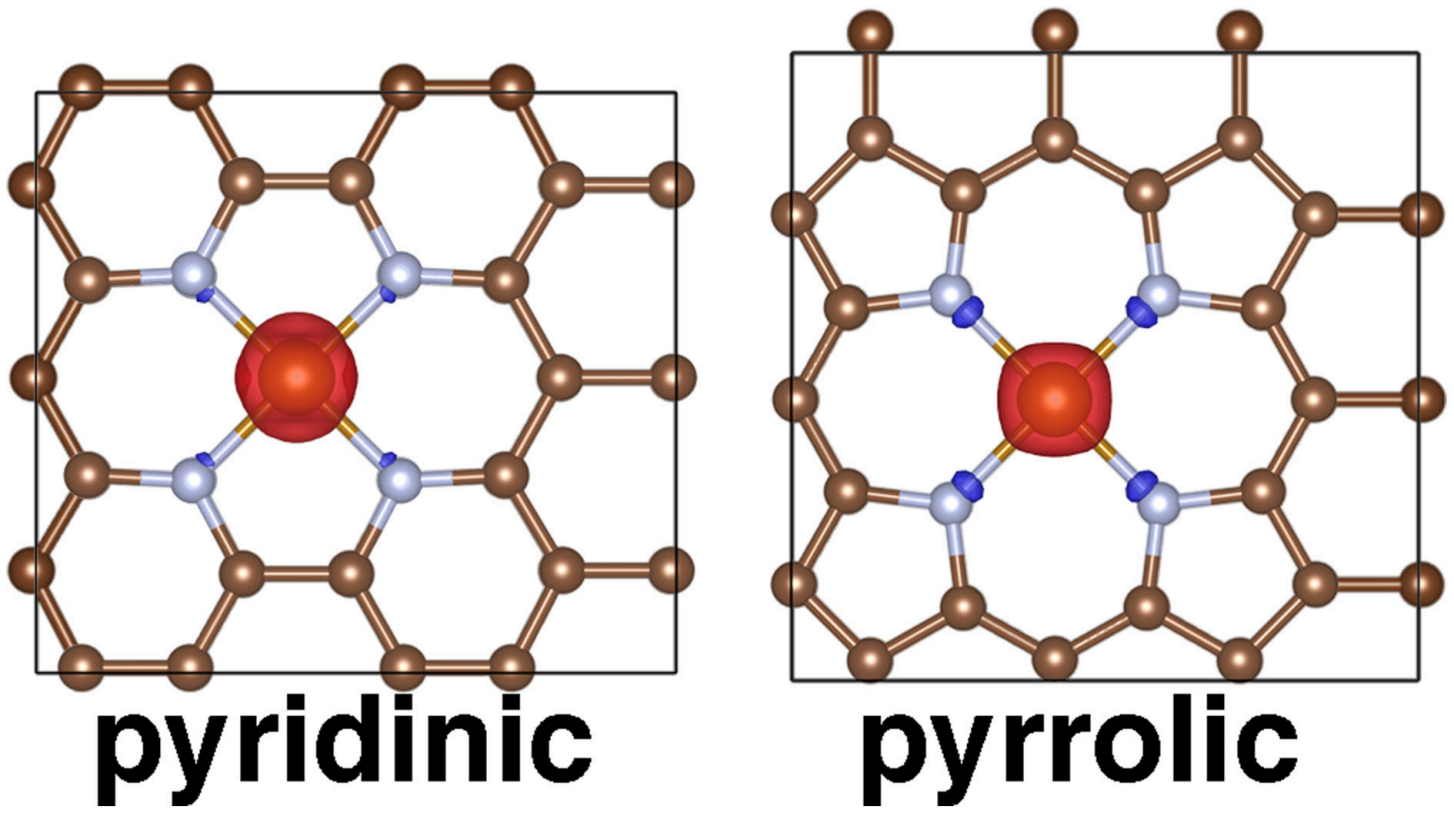
Periodic structures for pyridinic and pyrrolic periodic SAC models in the triplet state with spin density shown. Positive spin density is shown in red and negative spin density is shown in blue, with an isosurface value of 0.03 *e*/Bohr^3^. All structures are shown in ball and stick representation with carbon in brown, nitrogen in light blue, and iron in orange.

We validated our choice of finite SAC flakes by comparing to periodic models of both pyridinic and pyrrolic SAC active sites (Figure 9). We focused on the triplet intermediate spin state favored both in our finite models and in experiment (Liu et al., 2017). Shorter Fe-N 1.91 Å vs. 1.97 Å Fe-N bond lengths are observed for the pyridinic model than for the pyrrolic models, consistent with the finite models. It is more straightforward to localize the magnetic moment to the metal in these periodic systems than was observed for the molecular models (Supplementary Table 10 and Supplementary Figure 7). For the triplet cases, spin density is nearly exclusively observed on the metal center (Figure 9). In both pyridinic and pyrrolic periodic models, use of the hybrid functional leads to less electron density on the Fe center than when a GGA is employed, consistent with prior observations (Gani and Kulik, 2016; Zhao and Kulik, 2018) (Supplementary Table 10).

**Figure 10:**
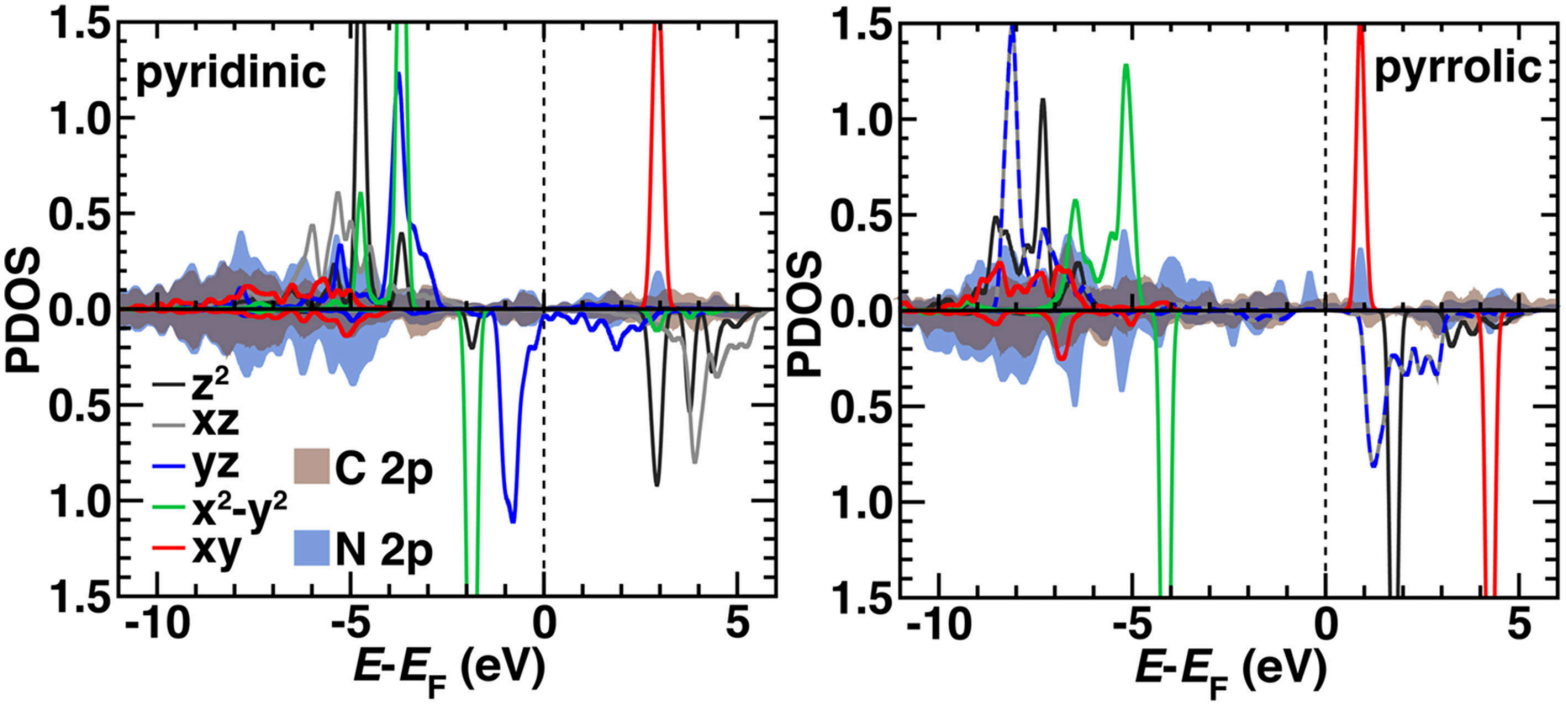
The HSE06 projected density of states (PDOS) for spin up **(Top)** and down (**Bottom**, reflected curves) triplet pyridinic **(Left)** and pyrrolic **(Right)** SAC models. The 3*d* Fe orbital PDOS are shown as indicated in inset legends as solid curves except for *yz* and *xz* in the pyrrolic case, which are shown as dashed lines due to their degeneracy. The average PDOS for a 2*p* orbital from C (brown) or N (dark blue) are shown as translucent shaded regions. All energy levels (in eV) are aligned to the Fermi level (*E*_F_), which is shown as a vertical dashed line. Some *d* levels have been truncated by the *y*-axis range to be able to compare to the broader C 2*p* and N 2*p* features.

We compared the electronic structure of the pyridinic and pyrrolic SAC active site models by determining the projected density of states (PDOS) decomposed by N 2*p*, C 2*p*, and Fe 3*d* contributions with the HSE06 hybrid functional (Figure 10). Qualitatively, the occupied orbitals and symmetries confirm observations made on the finite graphene flake models with PBE0, which may be expected if short range effects dominate as HSE06 and PBE0 both incorporate 25% exchange in the short range mixed with pure PBE exchange. That is, the pyridinic system again has singly occupied *d*_*z*_^2^ and *d*_*xz*_ orbitals and no occupation of the *d*_*xy*_ state. The pyrrolic system also differs from the pyridinic by having degenerate *d*_*xz*_ and *d*_*yz*_ states, consistent with the molecular flakes (Figure 10). Generally, agreement is more variable for other spin states, where convergence of the magnetic state is sensitive to starting conditions in the periodic calculation (Supplementary Figures 8–9). In the pyridinic case, *d*_*yz*_ states span the Fermi level, whereas the 3*d* states are well separated in the pyrrolic SAC (Figure 10). The pyrrolic 3*d* states also mix more deeply into the C and N 2*p* bands, whereas in the pyridinic case, most 3*d* states sit at the top of the occupied C/N 2*p* bands (Figure 10). Overall, these observations support the use of finite models at higher levels of theory for consistent modeling, due to the unique challenges of modeling periodic systems with such methods (Janet et al., 2017). More analysis in larger supercells with variable graphene defects will be necessary in future work to strengthen this conclusion.

## Conclusions and Outlook

We have presented an overview of the effect of computational model choice on the properties of octahedral transition metal complexes and emergent single atom catalyst (SAC) materials made from Fe centers in N-doped graphene. The octahedral transition metal complexes chosen mimic the ligand field environment observed in the SAC models but remain tractable for study with multi-reference wavefunction theory. Observations from the hexa-aqua and hexa-ammine complex studies revealed that spin state ordering of mid-row complexes could be sensitive both to the IPEA shift chosen and whether an extended active space was used, whereas late and early transition metals were far less sensitive. Then using these extended active space CASPT2 results as a benchmark, we observed that nearly all transition metal complexes benefitted from increased HF exchange. In fact, errors with respect to exchange fraction chosen monotonically decreased from 0 to 40% but with no improvement for higher exchange fractions. The 40% exchange hybrid results had comparable accuracy to the more computationally demanding double hybrid PBE0-DH.

The HF exchange tuning study confirmed the comparable behavior of Fe(II) complexes with ammonia, pyridine, and pyrrole ligands due to the overriding role of the metal, oxidation state, and ligand connecting atom in determining functional sensitivity. Comparison to CASPT2 results on the hexa-ammine system motivated us to propose higher exchange fractions (ca. 40% rather than 25% in PBE0) to be essential to counteract the low-spin bias in semi-local DFT. Using these benchmarks, we then evaluated the effect of DFT functional tuning on finite graphitic SAC models with pyridine or pyrrole nitrogen atoms. In these square planar SAC geometries, rigid structures compressed the Fe-N bond length, reducing exchange sensitivity of spin state ordering but otherwise confirming the observations in the octahedral complexes (i.e., favoring higher spin configurations and destabilizing singlet states). We observed that at the recommended higher exchange fractions, the formation of an oxo intermediate from N_2_O became unfavorable at the pyrrolic SAC active site. We also observed changes in spin state ordering of the most stable oxo intermediates. Thus, incorporation of exchange can alter predictions of reactivity at SAC active sites. Finally, we confirmed that these observations were not sensitive to choice of a finite SAC model by comparing to periodic SAC models where similar electron configurations were observed.

Overall, predictions of reactivity and spin state ordering are highly sensitive to the functional employed. We have shown that this sensitivity is broadly transferable across different ligand environments as long as the metal and direct ligating atom are kept constant. This observation can be leveraged to obtain DFT functional performance on smaller models where correlated WFT is tractable. Additionally, smaller flake models may be amenable to direct WFT calculation with methods not covered in this work. Spin state exchange sensitivity can be expected to be depressed in cases where the SAC is fully rigid and prevents expansion of the metal-ligand bond when the spin state changes. The highly ordered, symmetric cases here are expected to be the limit in this rigidity argument, and more disordered SAC models or more flexible active sites (e.g., graphitic carbon nitride) are expected to be more sensitive. Still, some outstanding challenges remain in understanding the extent to which spin arising on the graphene itself could be physical and also impart reactivity or whether it arises due to increased static correlation error for hybrid DFT. Furthermore, in periodic simulations with larger supercells than studied in this work it will become essential to employ range-separated hybrids with HF exchange only in the short range, and the comparison to CASPT2 results to range-separated hybrid tuning will be essential here. Overall, modeling in SACs will continue to benefit from this multi-level approach in assessing method accuracy and sensitivity and how method choice impacts predictions of active site geometry and reactivity.

## Author Contributions

HK designed the research. FL, TY, JY, EX, and AB carried out the research. FL, TY, JY, and HK wrote and revised the manuscript.

### Conflict of Interest Statement

The authors declare that the research was conducted in the absence of any commercial or financial relationships that could be construed as a potential conflict of interest.

## Footnotes

^1^OpenMolcas. Available online at: https://gitlab.com/Molcas/OpenMolcas.git (accessed June 6, 2018).
