# Supplementary material for: Bridging the Homogeneous-Heterogeneous Divide: Modeling Spin for Reactivity in Single Atom Catalysis
Source: Front Chem. 2019 Apr 16;7:219. doi: 10.3389/fchem.2019.00219 (PMC6476907; doi:10.3389/fchem.2019.00219)
Supplement: Supplementary file 1 [file Data_Sheet_1.PDF]

Supplementary Material

Bridging the homogeneous-heterogeneous divide: modeling spin and reactivity in single atom catalysis

Fang Liu[1], Tzuhsiung Yang[1], Jing Yang[1], Eve Xu[1], Akash Bajaj[1,2], Heather J. Kulik[1,*]

*** Correspondence:** Heather J. Kulik: hjkulik@mit.edu

Supplementary Table 1. High-spin (HS) /low-spin (LS) splitting energies (kcal/mol) of hexa-aqua and hexa-ammine complexes computed with CASPT2 in standard and extended active spaces, and the ground state (GS) spin state based on the result of the extended active space. An IPEA shift of 0.5 a.u. is used.

metal	n_{3d}*	transtion	$[M(H_2O)_6]^{n+}$				$[Mn(NH_3)_6]^{n+}$			
			ΔE standard	ΔE extended	Diff.	GS	ΔE standard	ΔE extended	Diff.	GS
Ti^{II}	2	HS-LS	-22.6	-22.09	0.51	HS	-23.27	-29.37	-6.1	HS
V^{III}	2	HS-LS	-28.59	-28.87	-0.28	HS	-30.38	-30.22	0.16	HS
V^{II}	3	HS-LS	-37.75	-37.01	0.74	HS	-42.18	-39.5	2.68	HS
Cr^{III}	3	HS-LS	-43.83	-40.57	3.26	HS	-44.83	-52.67	-7.84	HS
Cr^{II}	4	HS-LS	-57.91	-54.31	3.6	HS	-57.31	-48.27	9.04	HS
		HS-IS	-27.33	-28.8	-1.47		-26.14	-23.72	2.42	
Mn^{III}	4	HS-LS	-52.92	-53.38	-0.46	HS	-57.93	-52.91	5.02	HS
		HS-IS	-24.92	-21.97	2.95		-19	-17.88	1.12	
Mn^{II}	5	HS-LS	-78.71	-71.78	6.93	HS	201.34	-62.76	**-264.1**	HS
		HS-IS	-53.67	-47.36	6.31		213.71	-63.11	**-276.82**	
Fe^{III}	5	HS-LS	-56.17	-54.44	1.73	HS	-21.44	-21.18	0.26	HS
		HS-IS	-34.91	-39.1	-4.19		-22.81	-21.74	1.07	
Fe^{II}	6	HS-LS	-58.35	-56.83	1.52	HS	-27.88	-31.12	-3.24	HS
		HS-IS	-42.54	-39.96	2.58		-33.93	-34.8	-0.87	
Co^{III}	6	HS-LS	-23.64	-19.38	4.26	HS	34.85	28.36	-6.49	LS
		HS-IS	-12.81	-14.29	-1.48	HS	6.87	4.05	-2.82	
Co^{II}	7	HS-LS	-33.2	-45.44	**-12.24**	HS	-21.36	-21.45	-0.09	HS
Ni^{III}	7	HS-LS	1.32	1.81	0.49	LS	40.86	38.05	-2.81	LS
Ni^{II}	8	HS-LS	-40.29	-43.16	-2.87	HS	-20.13	-34.85	**-14.72**	HS
Cu^{III}	8	HS-LS	-7.33	-8.74	-1.41	HS	20.36	16.07	-4.29	LS

*Number of 3d electrons for the metal cation

Supplementary Table 2. Spin-state ordering calculated with modified PBE0 functional with different portion of HF exchange (i.e., $\Delta E_{\text{H-L/H-I}}$ in kcal) for hexa-aqua and hexa-ammine complexes with a metal center that has 3 to 7 3d electrons. For the 4, 5, and 6 d-electron cases, the transition corresponds to H-I rather than H-L.

	$\Delta E_{\text{H-L}}$ or $\Delta E_{\text{H-I}}$									$\frac{\partial \Delta E}{\partial a_{\text{HF}}}$	R^2
a_{HF}	0	0.1	0.2	0.3	0.4	0.5	0.6	0.8	1.0		
				Hexa-ammine							
V^{2+}	-45.2	-46.8	-48.4	-50.0	-51.4	-52.8	-45.2	-46.8	-48.4	-15.10	0.999
Cr^{3+}	-55.0	-57.1	-58.8	-60.6	-62.1	-63.6	-55.0	-57.1	-58.8	-17.02	0.996
Cr^{2+}	-13.6	-16.7	-19.5	-21.9	-24.1	-26.0	-13.6	-16.7	-19.5	-24.92	0.992
Mn^{3+}	-1.8	-5.9	-9.8	-13.3	-16.6	-19.6	-1.8	-5.9	-9.8	-35.63	0.997
Mn^{2+}	-23.8	-29.4	-34.5	-39.2	-43.5	-47.4	-23.8	-29.4	-34.5	-47.21	0.996
Fe^{3+}	2.6	-3.2	-8.8	-14.4	-20.0	-25.7	2.6	-3.2	-8.8	-56.47	1.000
Fe^{2+}	-9.1	-14.5	-19.6	-24.2	-28.4	-32.1	-9.1	-14.5	-19.6	-46.05	0.995
Co^{3+}	19.7	14.4	9.3	4.6	0.0	-4.6	19.7	14.4	9.3	-48.31	0.999
Co^{2+}	1.2	-5.0	-10.8	-16.2	-21.1	-25.5	1.2	-5.0	-10.8	-53.61	0.996
Ni^{3+}	33.5	27.9	22.9	18.3	13.9	9.6	33.5	27.9	22.9	-47.46	0.998
**MAE**	**16.6**	**14.0**	**11.5**	**11.4**	**7.9**	**9.2**	**13.8**	**18.6**	**23.0**		
				Hexa-aqua							
V^{2+}	-33.0	-36.0	-38.7	-41.1	-43.4	-45.5	-33.0	-36.0	-38.7	-24.77	0.996
Cr^{3+}	-31.9	-36.6	-40.8	-44.7	-48.2	-51.4	-31.9	-36.6	-40.8	-38.77	0.995
Cr^{2+}	-19.9	-22.4	-24.6	-26.6	-28.5	-30.2	-19.9	-22.4	-24.6	-20.49	0.995
Mn^{3+}	-9.4	-12.9	-16.1	-19.2	-22.0	-24.7	-9.4	-12.9	-16.1	-30.56	0.998
Mn^{2+}	-28.9	-32.5	-37.7	-38.7	-41.3	-43.8	-28.9	-32.5	-37.7	-29.16	0.966
Fe^{3+}	-7.5	-20.6	-26.2	-30.7	-35.7	-40.7	-7.5	-20.6	-26.2	-61.70	0.952
Fe^{2+}	-18.1	-21.1	-23.7	-26.1	-28.2	-30.1	-18.1	-21.1	-23.7	-23.99	0.994
Co^{3+}	-0.4	-2.4	-5.0	-8.0	-11.6	-15.5	-0.4	-2.4	-5.0	-30.30	0.986
Co^{2+}	-19.3	-22.7	-26.0	-29.1	-31.9	-34.5	-19.3	-22.7	-26.0	-30.38	0.998
Ni^{3+}	-6.9	-6.6	-6.7	-7.4	-9.1	-11.7	-6.9	-6.6	-6.7	-9.26	0.742
**MAE**	**15.5**	**11.6**	**8.8**	**9.3**	**6.3**	**6.4**	**7.9**	**11.3**	**14.7**		

Supplementary Table 3. Spin-state ordering (i.e., $\Delta E_{\text{H-L}}$ in kcal) calculated with modified PBE0 functional with different portion of HF exchange for hexa-aqua and hexa-ammine complexes with a metal center has 2 to 8 3d electrons. All the transition corresponds to H-L.

	$\Delta E_{\text{H-L}}$						$\frac{\partial \Delta E_{\text{H-L}}}{\partial a_{\text{HF}}}$	R^2
	0	0.1	0.2	0.3	0.4	0.5		
Hexa-ammine								
Ti^{2+}	-30.5	-32.4	-34.0	-35.5	-36.8	-38.1	-15.12	0.996
V^{3+}	-39.9	-41.8	-43.6	-45.2	-46.8	-48.4	-16.85	0.999
V^{2+}	-45.2	-46.8	-48.4	-50.0	-51.4	-52.8	-15.10	0.999
Cr^{3+}	-55.0	-57.1	-58.8	-60.6	-62.1	-63.6	-17.02	0.996
Cr^{2+}	-47.1	-51.2	-54.9	-58.2	-61.1	-63.8	-33.24	0.994
Mn^{3+}	-43.4	-48.7	-53.6	-58.1	-62.2	-65.9	-44.96	0.996
Mn^{2+}	-28.2	-36.7	-44.2	-50.8	-56.5	-61.8	-66.79	0.992
Fe^{3+}	14.3	4.9	-4.2	-13.0	-21.5	-29.6	-87.75	0.999
Fe^{2+}	1.6	-7.1	-15.2	-22.4	-28.8	-34.5	-72.20	0.994
Co^{3+}	47.9	39.2	31.4	23.8	16.4	9.3	-76.75	0.999
Co^{2+}	1.2	-5.0	-10.8	-16.2	-21.1	-25.5	-53.61	0.996
Ni^{3+}	33.5	27.9	22.9	18.3	13.9	9.6	-47.46	0.998
Ni^{2+}	2.6	-5.1	-13.0	-20.9	-28.6	-35.9	-77.55	1.000
Cu^{3+}	-12.2	-13.4	-14.8	-16.6	-18.6	-21.2	-17.80	0.982
Hexa-aqua								
Ti^{2+}	-27.3	-28.9	-30.3	-31.6	-32.9	-34.0	-13.36	0.995
V^{3+}	-31.2	-33.8	-36.0	-38.1	-39.9	-41.6	-20.67	0.994
V^{2+}	-33.0	-36.0	-38.7	-41.1	-43.4	-45.5	-24.77	0.996
Cr^{3+}	-31.9	-36.6	-40.8	-44.7	-48.2	-51.4	-38.77	0.995
Cr^{2+}	-52.6	-57.6	-62.1	-66.1	-69.8	-73.2	-41.10	0.995
Mn^{3+}	-11.3	-20.5	-28.9	-36.4	-43.2	-49.4	-76.09	0.995
Mn^{2+}	-50.3	-57.2	-63.4	-69.1	-74.3	-79.0	-57.36	0.995
Fe^{3+}	-18.2	-25.0	-32.2	-39.7	-47.3	-54.7	-73.45	1.000
Fe^{2+}	-34.7	-39.8	-44.7	-49.0	-53.0	-56.6	-43.85	0.996
Co^{3+}	-9.4	-11.1	-13.9	-17.6	-22.1	-27.1	-35.83	0.972
Co^{2+}	-19.3	-22.7	-26.0	-29.1	-31.9	-34.5	-30.38	0.998
Ni^{3+}	-6.9	-6.6	-6.7	-7.4	-9.1	-11.7	-9.26	0.742
Ni^{2+}	-30.6	-36.2	-41.7	-46.8	-51.4	-55.7	-50.40	0.997
Cu^{3+}	-10.9	-15.0	-19.4	-23.0	-27.1	-31.7	-41.07	0.999

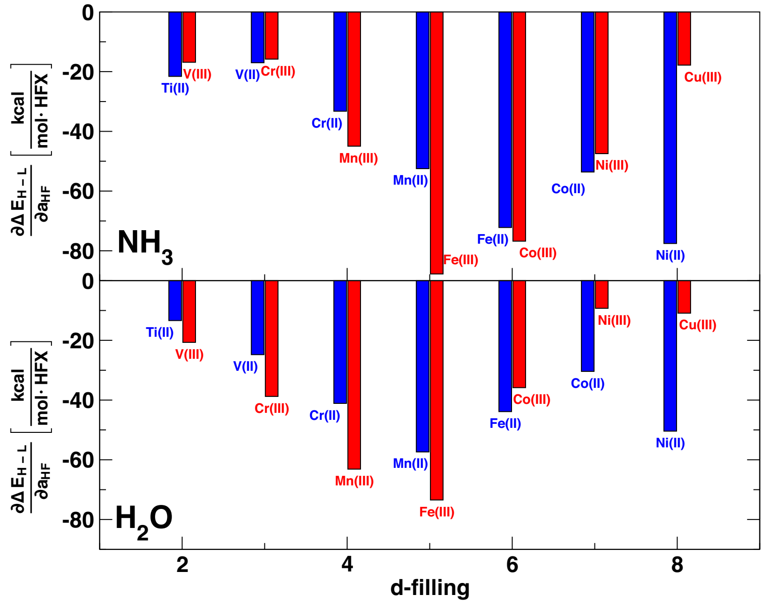

Supplementary Figure 1. Sensitivity of spin-state splitting with respect to HF exchange (i.e., $\partial\Delta E_{H\text{-}L}/\partial a_{HF}$, in kcal/mol·HFX) for hexa-aqua (top) and hexa-ammine (bottom) transition metal complexes. Both M(II) and M(III) complexes are shown grouped by their nominal *d* filling from 3 to 7 3*d* electrons for Ti(II) to Ni(III). Here the comparison is always HS-LS, which includes 4 electron differences for the 4, 5, 6 d-electron cases versus the main text figure which contains HS-IS cases for those cases.

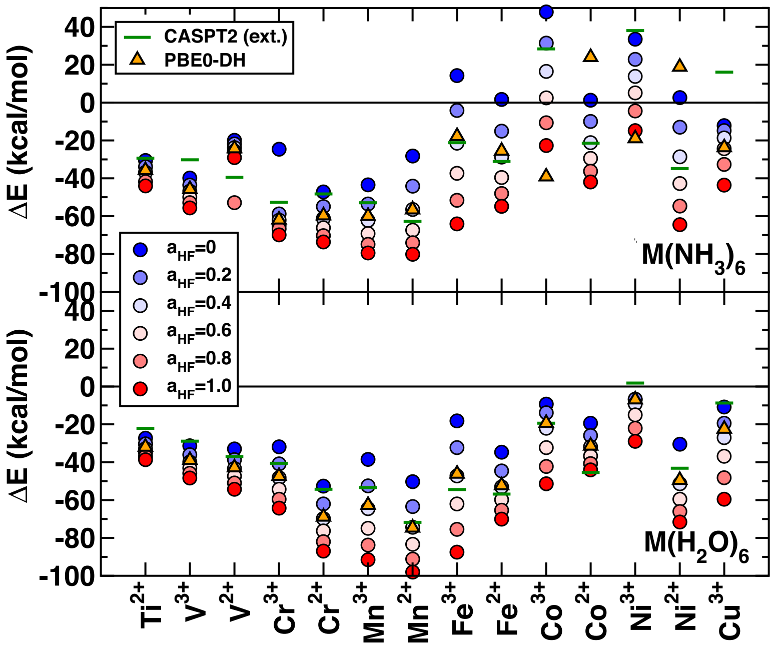

Supplementary Figure 2. Spin-state ordering (i.e., $\Delta E_{\text{H-L}}$ in kcal) calculated with modified PBE0 functional with different portion of HF exchange, CASPT2 with extended active site, and PBE0 based double hybrid (PBE0-DH) for hexa-aqua and hexa-ammine complexes whose metal center has 2 to 8 3d electrons. Here the comparison is always HS-LS, which includes 4 electron differences for the 4, 5, 6 d-electron cases versus the main text figure which contains HS-IS cases for those cases.

Supplementary Table 4. PBE0 and PBE0-DH spin state ordering (in kcal/mol) for octahedral complexes optimized with PBE0/def2-TZVP, as described in the main text.

Ligand	core	HS-LS		HS-IS	
		PBE0	PBE0-DH	PBE0	PBE0-DH
NH_3	Ti^{2+}	-34.73	-35.74		
	V^{3+}	-44.48	-45.77		
	V^{2+}	-38.13	-24.25		
	Cr^{3+}	-27.64	-61.86		
	Cr^{2+}	-56.58	-59.67	-20.14	-23.16
	Mn^{3+}	-55.91	-59.94	-11.73	-15.21
	Mn^{2+}	-47.59	-56.53	-36.92	-43.69
	Fe^{3+}	-8.64	-17.76	-11.60	-18.69
	Fe^{2+}	-18.87	-25.49	-21.76	-27.81
	Co^{3+}	27.52	23.98	7.17	-23.16
	Co^{2+}	-13.56	-18.93		
	Ni^{3+}	20.55	18.86		
	Ni^{2+}	-16.99	-23.67		
	Cu^{3+}	-15.65	-14.05		
H_2O	Ti^{2+}	-31.00	-31.89		
	V^{3+}	-37.09	-38.88		
	V^{2+}	-39.94	-42.79		
	Cr^{3+}	-42.80	-47.32		
	Cr^{2+}	-64.13	-68.84	-25.63	-28.48
	Mn^{3+}	-55.63	-62.62	-17.68	-21.73
	Mn^{2+}	-66.33	-74.63	-37.22	-42.84
	Fe^{3+}	-35.97	-46.14	-18.97	-35.78
	Fe^{2+}	-46.90	-52.24	-24.91	-29.36
	Co^{3+}	-15.62	-19.30	-6.35	-10.33
	Co^{2+}	-27.54	-31.34		
	Ni^{3+}	-6.97	-6.91		
	Ni^{2+}	-44.27	-49.42		
	Cu^{3+}	-21.15	-22.40		
pyridine	Fe^{3+}	-23.26			
	Fe^{2+}	-15.01			
pyrrole	Fe^{3+}	-11.90			
	Fe^{2+}	-29.91			

Supplementary Table 5. Bond lengths of Fe(II)/Fe(III) octahedral complexes optimized with PBE0/def2-TZVP, as described in the main text.

metal center	ligand	spin state	$Fe-N_1$	$Fe-N_2$	$Fe-N_3$	$Fe-N_4$	$Fe-N_5$	$Fe-N_6$
Fe^{2+}	pyridine	HS	2.292	2.297	2.297	2.292	2.311	2.310
Fe^{2+}	pyridine	LS	2.081	2.153	2.153	2.081	2.098	2.098
Fe^{3+}	pyridine	HS	2.194	2.218	2.217	2.194	2.219	2.220
Fe^{3+}	pyridine	LS	2.051	2.113	2.120	2.061	2.079	2.066
Fe^{2+}	pyrrole	HS	2.271	2.272	2.311	2.311	2.382	2.382
Fe^{2+}	pyrrole	LS	2.113	2.112	2.114	2.114	2.115	2.115
Fe^{3+}	pyrrole	HS	2.156	2.157	2.155	2.155	2.157	2.157
Fe^{3+}	pyrrole	LS	2.024	2.023	2.022	2.022	2.025	2.024
Fe^{2+}	NH_3	HS	2.274	2.278	2.278	2.293	2.293	2.274
Fe^{2+}	NH_3	IS	2.071	2.333	2.333	2.195	2.195	2.071
Fe^{2+}	NH_3	LS	2.084	2.084	2.084	2.085	2.085	2.084
Fe^{3+}	NH_3	HS	2.199	2.199	2.199	2.199	2.199	2.199
Fe^{3+}	NH_3	IS	2.061	2.061	2.061	2.293	2.293	2.061
Fe^{3+}	NH_3	LS	2.048	2.048	2.048	2.047	2.048	2.048
Fe^{2+}	H_2O	HS	2.167	2.113	2.173	2.167	2.113	2.173
Fe^{2+}	H_2O	IS	2.121	2.163	2.035	2.120	2.164	2.035
Fe^{2+}	H_2O	LS	2.032	2.001	2.032	2.032	2.001	2.032
Fe^{3+}	H_2O	HS	2.053	2.033	2.067	2.053	2.033	2.067
Fe^{3+}	H_2O	IS	1.895	2.081	1.992	1.894	2.081	1.991
Fe^{3+}	H_2O	LS	1.974	1.896	1.924	1.974	1.896	1.924

Supplementary Table 6. Bond lengths of FeN_4C_{10} and FeN_4C_{12} graphene SAC flake models optimized with PBE0/def2-TZVP, as described in the main text.

Complex	multiplicity	$Fe-N_1$	$Fe-N_2$	$Fe-N_3$	$Fe-N_4$
FeN_4C_{10}	singlet	1.894	1.893	1.893	1.894
FeN_4C_{10}	triplet	1.896	1.895	1.895	1.896
FeN_4C_{10}	quintet	1.908	1.907	1.907	1.908
FeN_4C_{12}	singlet	1.969	1.968	1.969	1.967
FeN_4C_{12}	triplet	1.954	1.952	1.954	1.952
FeN_4C_{12}	quintet	1.960	1.956	1.958	1.958

Supplementary Table 7. PBE0 spin state ordering for geometry optimized SAC models.

Complex	multiplicity	relative energy (kcal/mol)
FeN_4C_{10}	singlet	4.20
FeN_4C_{10}	triplet	0.00
FeN_4C_{10}	quintet	12.40
FeN_4C_{12}	singlet	5.68
FeN_4C_{12}	triplet	0.00
FeN_4C_{12}	quintet	12.60

Supplementary Table 8. Mulliken spins of iron metal (M), coordinating nitrogen atoms (N1-N4), and all remaining atoms (Other) for the pyridinic N4C10 graphene flake SAC models without (left) and with formed oxo species (right).

	N4C10						N4C10-oxo						
% HF	M	N1	N2	N3	N4	Other	M	N1	N2	N3	N4	O	Other
	singlet						singlet						
0	0.0	0.0	0.0	0.0	0.0	0.0	0.0	0.0	0.0	0.0	0.0	0.0	0.0
10	0.0	0.0	0.0	0.0	0.0	0.0	0.0	0.0	0.0	0.0	0.0	0.0	0.0
20	0.0	0.0	0.0	0.0	0.0	0.0	0.0	0.0	0.0	0.0	0.0	0.0	0.0
30	0.0	0.0	0.0	0.0	0.0	0.0	0.0	0.0	0.0	0.0	0.0	0.0	0.0
40	0.0	0.0	0.0	0.0	0.0	0.0	0.0	0.0	0.0	0.0	0.0	0.0	0.0
50	0.0	0.0	0.0	0.0	0.0	0.0	0.0	0.0	0.0	0.0	0.0	0.0	0.0
	triplet						triplet						
0	2.2	-0.1	-0.1	-0.1	-0.1	0.0	1.2	0.0	0.0	0.0	0.0	0.8	0.0
10	2.2	-0.1	-0.1	-0.1	-0.1	0.0	1.3	0.0	0.0	0.0	0.0	0.7	0.0
20	2.2	-0.1	-0.1	-0.1	-0.1	0.0	1.4	0.0	0.0	0.0	0.0	0.6	0.0
30	2.2	-0.1	-0.1	-0.1	-0.1	0.0	1.5	0.0	0.0	0.0	0.0	0.7	-0.1
40	2.2	-0.1	-0.1	-0.1	-0.1	0.0	1.6	0.0	0.0	0.0	0.0	0.6	-0.1
50	2.2	-0.1	-0.1	-0.1	-0.1	0.0	1.8	-0.1	-0.1	-0.1	-0.1	0.5	-0.1
	quintet						quintet						
0	2.2	0.0	0.0	0.0	0.0	1.8	1.4	0.0	0.0	0.0	0.0	0.9	1.7
10	2.2	0.0	0.0	0.0	0.0	1.8	1.4	0.0	0.0	0.0	0.0	0.9	1.7
20	2.2	0.0	0.0	0.0	0.0	1.8	1.5	0.0	0.0	0.0	0.0	0.8	1.7
30	2.2	0.0	0.0	0.0	0.0	1.8	1.6	0.0	0.0	0.0	0.0	0.7	1.7
40	2.2	0.0	0.0	0.0	0.0	1.8	1.7	0.0	0.0	0.0	0.0	0.6	1.7
50	2.1	0.0	0.0	0.0	0.0	1.9	1.9	0.0	0.0	0.0	0.0	0.4	1.7

Supplementary Table 9. Mulliken spins of iron metal (M), coordinating nitrogen atoms (N1-N4), and all remaining atoms (Other) for the pyrrolic N4C12 graphene flake SAC models without (left) and with formed oxo species (right).

	N4C12						N4C12-oxo						
% HF	M	N1	N2	N3	N4	Other	M	N1	N2	N3	N4	O	Other
	singlet						singlet						
0	-1.4	0.1	0.1	0.1	0.1	1.0	0.0	0.0	0.0	0.0	0.0	0.0	0.1
10	-1.9	0.2	0.2	0.2	0.2	1.3	0.0	0.0	0.0	0.0	0.0	0.0	0.0
20	-2.0	0.2	0.2	0.2	0.2	1.3	0.1	0.0	0.0	-0.1	0.0	-0.1	0.0
30	-2.1	0.2	0.2	0.2	0.2	1.4	0.1	0.0	0.1	-0.1	0.0	-0.1	0.0
40	-2.1	0.2	0.2	0.2	0.2	1.3	-0.1	0.0	0.1	-0.1	0.0	0.1	0.0
50	-2.1	0.2	0.2	0.2	0.2	1.3	0.0	0.0	0.0	-0.1	0.0	0.0	0.0
	triplet						triplet						
0	2.4	0.0	0.0	0.0	0.0	-0.2	1.1	0.0	0.0	0.0	0.0	0.8	0.2
10	2.6	-0.1	-0.1	-0.1	-0.1	-0.3	1.2	0.0	0.0	0.0	0.0	0.8	0.0
20	2.7	-0.1	-0.1	-0.1	-0.1	-0.4	1.3	0.0	0.0	0.0	0.0	0.8	0.0
30	2.8	-0.1	-0.1	-0.1	-0.1	-0.5	1.3	0.0	0.0	0.0	0.0	0.8	0.0
40	3.0	-0.1	-0.1	-0.1	-0.1	-0.6	1.3	0.0	0.0	0.0	0.0	0.7	-0.1
50	3.1	-0.1	-0.1	-0.1	-0.1	-0.7	1.1	0.0	0.0	0.0	0.0	1.0	-0.1
	quintet						quintet						
0	2.5	0.0	0.0	0.0	0.0	1.6	1.3	0.1	0.0	0.0	0.1	0.8	1.6
10	2.6	0.0	0.0	0.0	0.0	1.6	1.3	0.1	0.0	0.0	0.1	0.8	1.6
20	2.5	0.0	0.0	0.0	0.0	1.6	1.3	0.1	0.0	0.0	0.1	0.8	1.6
30	2.4	0.0	0.0	0.0	0.0	1.7	1.3	0.1	0.0	0.0	0.1	0.8	1.7
40	2.2	0.0	0.0	0.0	0.0	1.9	1.3	0.1	0.0	0.0	0.1	0.7	1.9
50	2.2	0.0	0.0	0.0	0.0	2.0	1.0	0.1	0.0	0.0	0.1	1.0	1.9

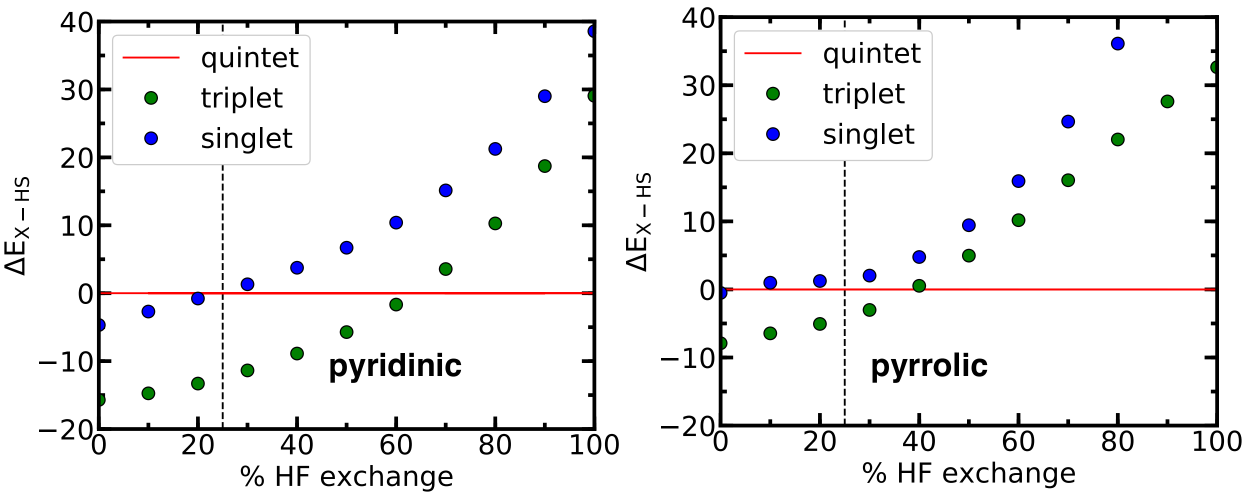

Supplementary Figure 3. Relative spin state energetics (in kcal/mol) for singlet (blue circles) or triplet (green circles) spin states with respect to quintet reference (red zero line) vs. % HF exchange

for pyridinic (FeN_4C_{10}, left) and pyrrolic (FeN_4C_{12}, right) SAC models. The 25% exchange in standard PBE0 is indicated as a vertical dashed line, and a full range of 0-100% HF is shown here instead of 0-50% as in the main text.

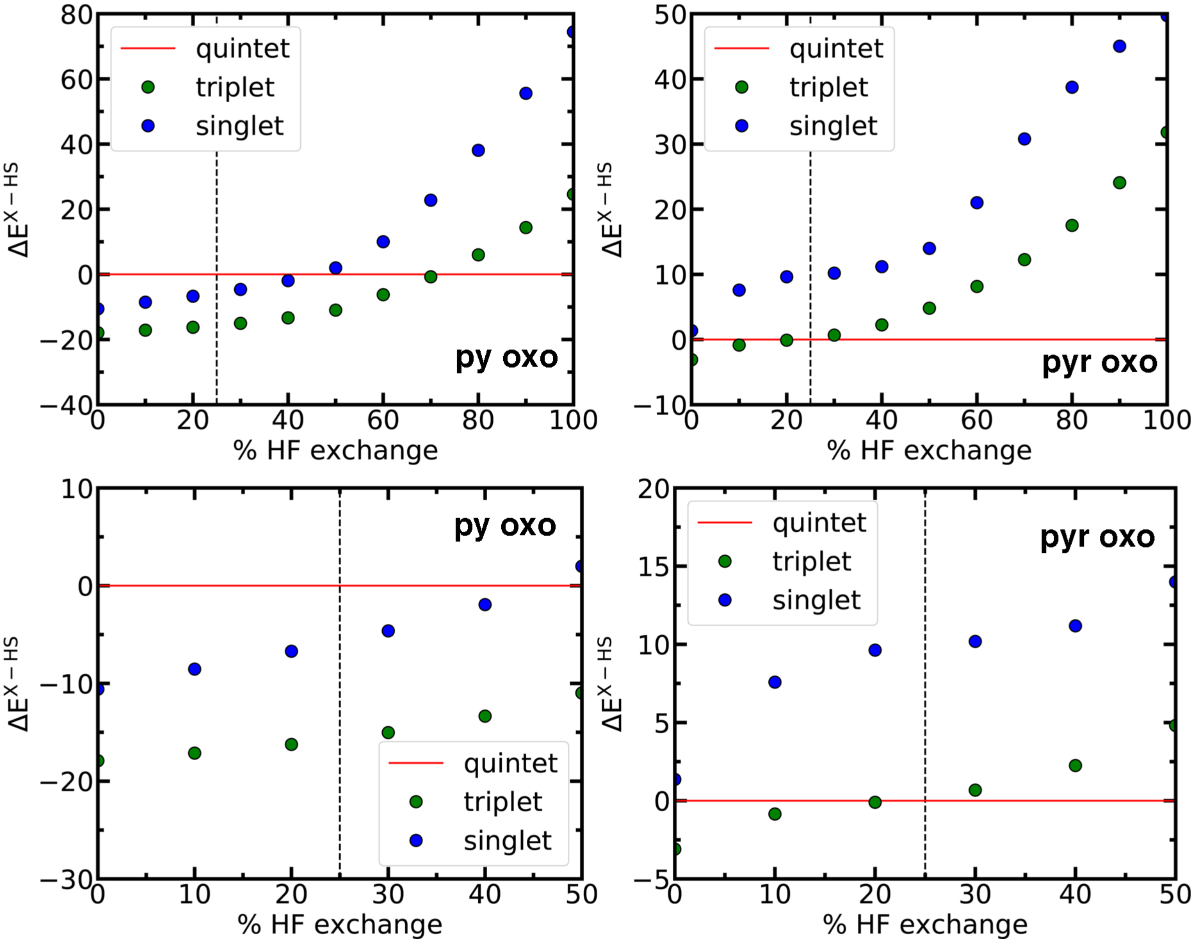

Supplementary Figure 4. Relative spin state energetics (in kcal/mol) for singlet (blue circles) or triplet (green circles) spin states with respect to quintet reference (red zero line) vs. % HF exchange for pyridinic ($FeN_4C_{10,}$ py oxo shown left) and pyrrolic (FeN_4C_{12}, pyr oxo shown right) SAC models. The 25% exchange in standard PBE0 is indicated as a vertical dashed line, and a full range of 0-100% HF is shown at top with 0-50% shown below in each case to highlight the most commonly applied exchange fraction results.

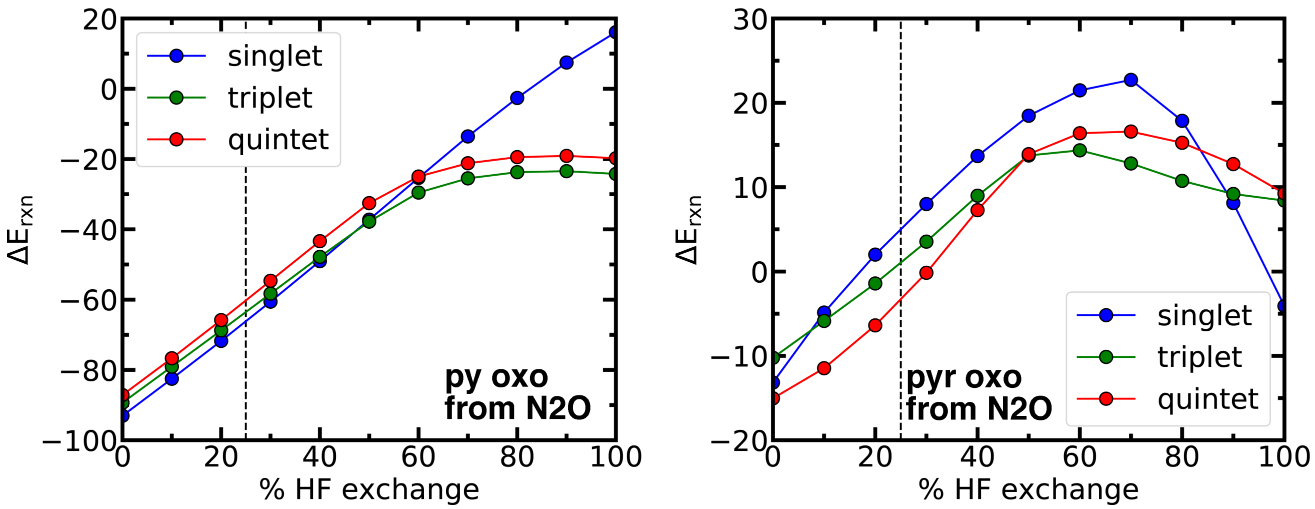

Supplementary Figure 5. Reaction energetics (ΔE_{rxn}, in kcal/mol) for oxo formation from N_2O oxidant vs. % HF exchange for pyridinic (FeN_4C_{10}, left, py oxo) and pyrrolic (FeN_4C_{12}, right, pyr oxo) SAC models. In each case, singlet (blue circles), triplet (green circles), and quintet (red circles) oxo formation energies are shown. The 25% exchange in standard PBE0 is indicated as a vertical dashed line. This differs from the main text figure by showing results for 0-100% exchange.

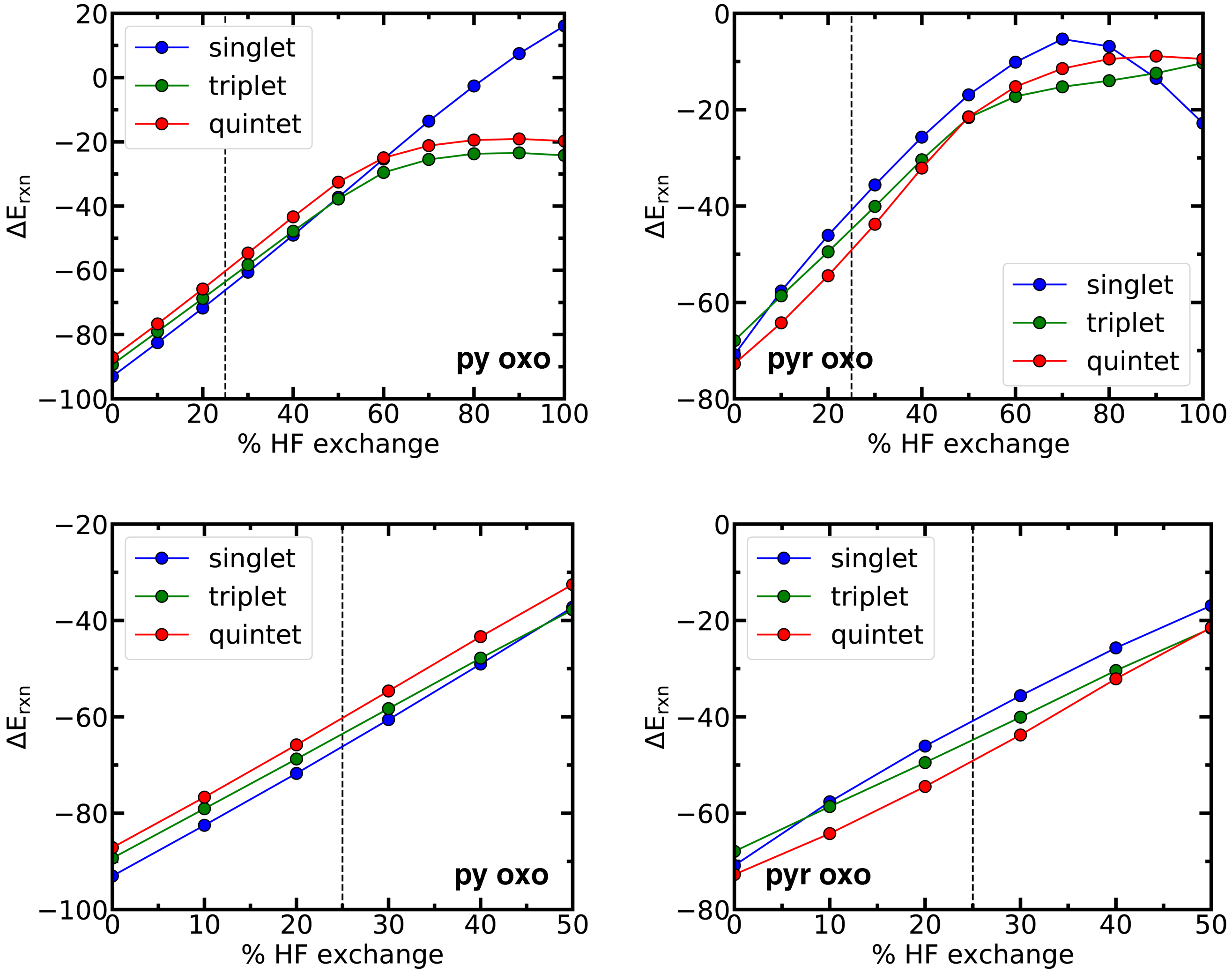

Supplementary Figure 6. Reaction energetics (ΔE_{rxn}, in kcal/mol) for oxo formation from O_2 vs. % HF exchange for pyridinic (FeN_4C_{10}, left, py oxo) and pyrrolic (FeN_4C_{12}, right, pyr oxo) SAC models. In each case, singlet (blue circles), triplet (green circles), and quintet (red circles) oxo formation energies are shown. The 25% exchange in standard PBE0 is indicated as a vertical dashed line. The top figures show the full 0-100% exchange range, whereas the bottom shows only 0-50% to highlight results for the most commonly applied exchange fractions.

Supplementary Table 10. PBE GGA and HSE06 periodic pyridinic and pyrrolic model spin states with 3d up and down electron count, Fe magnetization (in μ_B), and total energy (in Ry).

	PBE				HSE			
	pyridinic							
	E (Ry)	Fe mag	d↑	d↓	E (Ry)	Fe mag	d↑	d↓
singlet (CS)	-322.84459792	0.0	3.97	3.97	-322.33830997	0.0	3.18	3.18
singlet (OS)	-322.93240781	1.7	4.94	2.93	-322.33532489	1.7	4.23	2.18
triplet	-322.99077593	2.1	5.10	2.58	-322.41461639	2.0	4.30	2.01
quintet	-322.99873331	3.3	5.63	1.80	-322.40844033	3.4	4.93	1.08
	pyrrolic							

singlet (CS)	-345.56254968	-0.3	3.12	3.38	-345.01133366	0.0	3.16	3.16
singlet (OS)	-345.68077590	1.7	4.95	2.86	-345.06272471	1.7	4.18	2.17
triplet	-345.73821122	2.7	5.33	2.18	-345.13292332	2.8	4.52	1.44
quintet	-345.73718596	3.6	5.72	1.63	-345.13796231	4.0	4.96	0.66

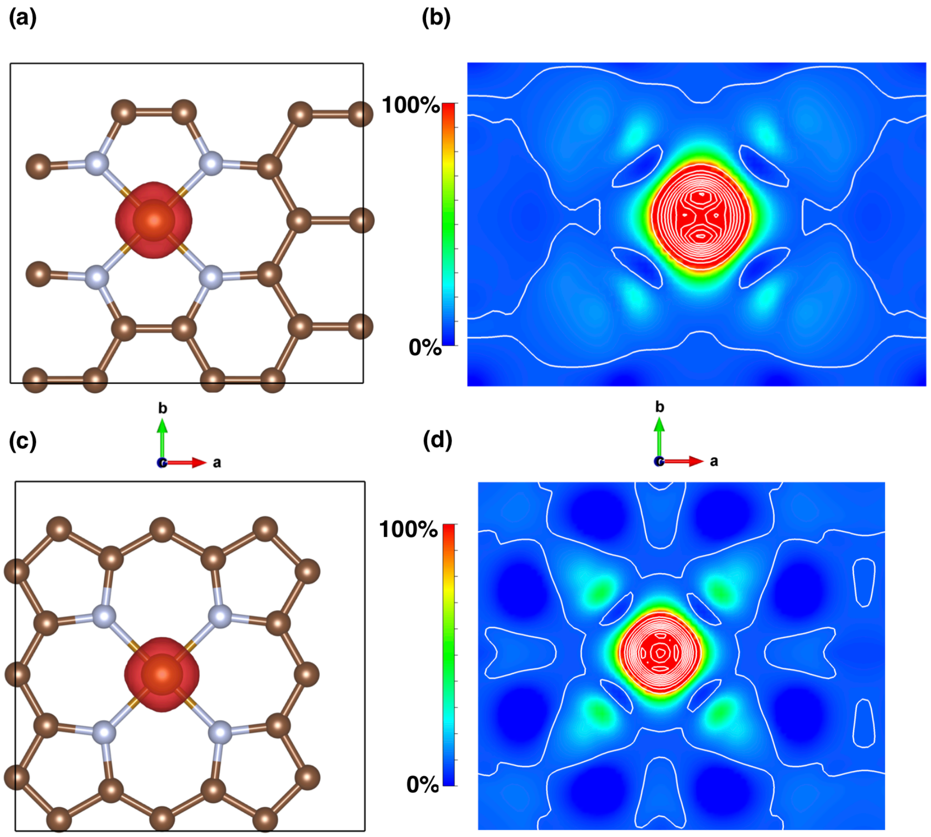

Supplementary Figure 7. (a) and (c): spin density of quintet state of Fe@pyridinic N and Fe@pyrrolic N system, respectively. (b) and (d) are the planar spin-up charge density distribution in the xy plane. The isosurface value for (a) and (c) are 0.033 and 0.035. The contour lines are in white color, and density is scale from 0% to 100% indicating the least spin-up to the most spin-up density. The spin-up electrons localized around Fe atom in Fe@pyridinic N system contain a symmetry that is very similar to dx^2-y^2 orbital but with higher density along y-axis as more contour lines align on this direction. On the other hand, Fe@pyrrolic N system Fe atom presents a dx^2-y^2-like profile but the distribution is even along x- and y-axis. The N atom in Fe@pyridinic N system presents a lower charge density than Fe@pyrrolic N. As a result, Fe@pyridinic N system contains a much more diffuse density profile rather than that of Fe@pyrrolic N system.

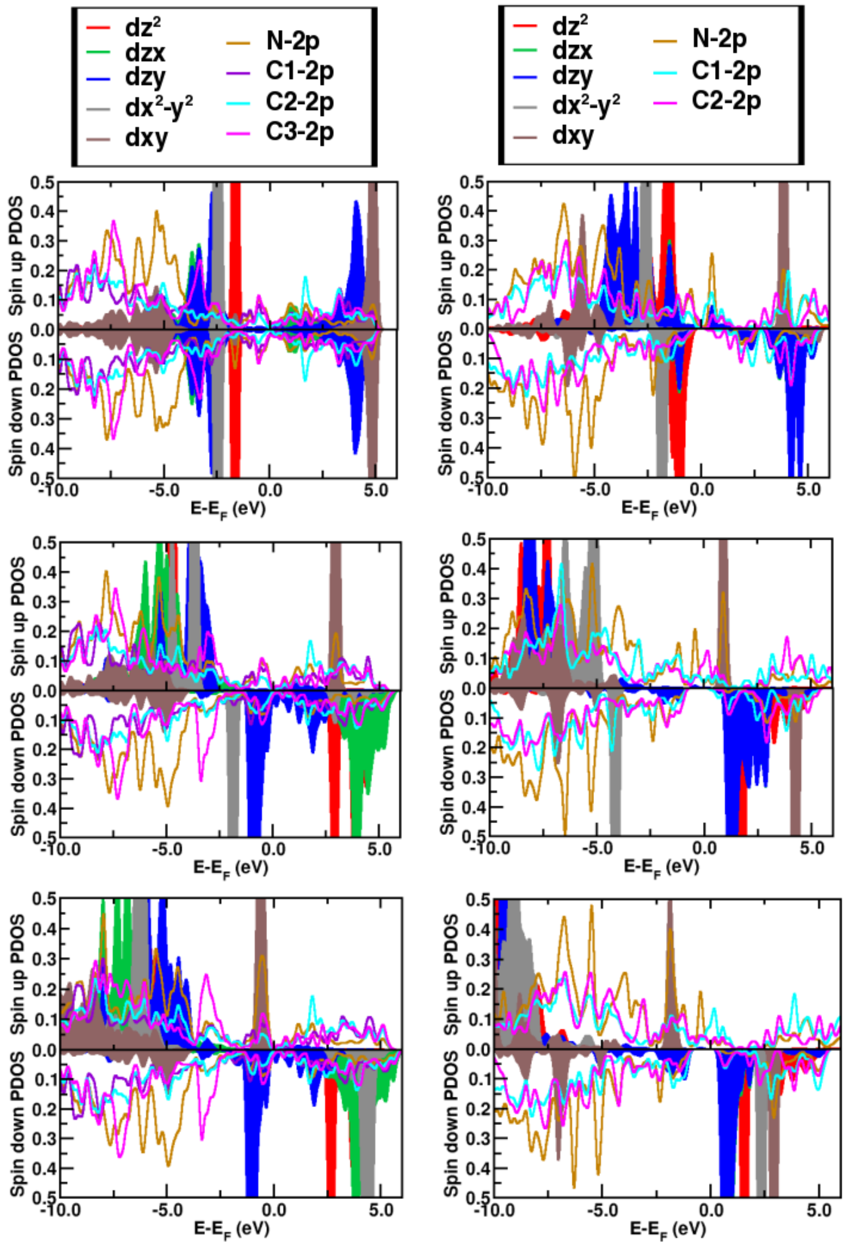

Supplementary Figure 8. Spin polarized projected density of states for Fe, N and C atoms with HSE. The left panel shows Fe@pyridinic N system at singlet (top), triplet (middle) and quintet (bottom) cases, whereas the right panel shows Fe@pyrrolic N system at singlet, triplet and quintet from top to bottom. The filled area indicates the Fe d-states, and the line features are N and C 2p total density of states. At the singlet ground state, the almost symmetric shape distribution and line features for all atoms species show that the system is spin-unpolarized. We categorize the carbon atoms into different groups based on the distance away from Fe center, with "C1" being the closest and "C3" being the furthest (see Supplementary Figure 9). In Fe@pyridinic N system, three types of carbon atom species are found, and two for Fe@pyrrolic N system.

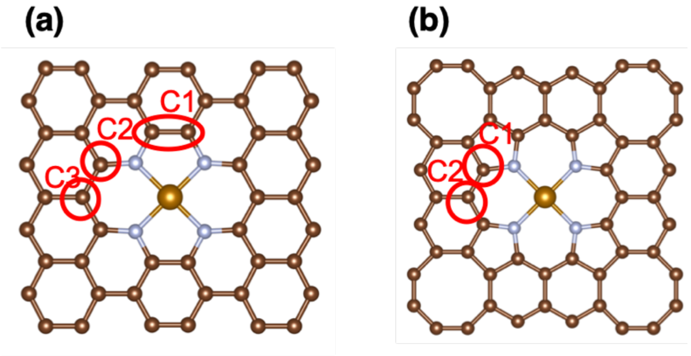

Supplementary Figure 9. Ball-and-stick picture showing different carbon species near Fe atom. (a) Fe@pyridinic N system (b) Fe@pyrrolic N system.
